# Synthesis of the Natural Product 1,3-Dibehenyl-2-ferulyl Glyceride (*Aquilaria malaccensis*) and Its Derivatives

**DOI:** 10.3390/molecules31101593

**Published:** 2026-05-09

**Authors:** Alexis de Jesús Sánchez-Esparza, Margarita Cantú-Reyes, Amira Jalil Fragoso-Medina, Antonio Nieto-Camacho, Miguel Ángel Ortega-Ruiz, Oscar Yael Osorio-Godínez, Alejandra Chávez-Riveros, Gabriel Cuevas, David Atahualpa Contreras-Cruz

**Affiliations:** 1 Instituto de Química, Universidad Nacional Autónoma de México, Ciudad de México C.P. 04510, Mexico; alexis.qfb.sanchezesparza@gmail.com (A.d.J.S.-E.);; 2 Facultad de Estudios Superiores Zaragoza, Universidad Nacional Autónoma de México, Ciudad de México C.P. 09230, Mexico; cantureyes.margarita@zaragoza.unam.mx (M.C.-R.);; 3 Instituto de Física, Universidad Nacional Autónoma de México, Ciudad de México C.P. 04510, Mexico; amira@fisica.unam.mx

**Keywords:** synthesis, 1,3-dibenhenyl-2-ferulyl glyceride, *Aquilaria malaccensis*, natural product, cosmetical industry

## Abstract

The first optimal and suitable synthetic route was developed to obtain the natural product 1,3-dibehenyl-2-ferulyl glyceride (**1**) in four reaction steps with an overall yield of 23%. Additionally, a collection of ten derivatives of the natural product was prepared with similar reaction yields. Compound **19** showed potent antioxidant activity through the DPPH assay with an inhibition higher than 95% (IC_50_ 17.58 ± 0.45 µM). Furthermore, its characterization using ^1^H NMR, ^13^C NMR, IR and MS techniques is presented. This work is relevant because it addresses the need for a natural product with very low availability and that comes from an endangered species. The compounds feature a conjugated lipid–drug structure, with cinnamic, caffeic and ferulic acids in the pharmacologically active portion, making its evaluation in biological tests of great interest.

## 1. Introduction

One of the common characteristics of living beings is that they share pathways by which they produce certain essential chemical substances. Compounds essential for the survival and well-being of the organism are called primary metabolites [1]. In addition to primary metabolites, organisms produce other non-essential chemicals through unconventional metabolic pathways. The compounds resulting from these metabolic processes are known as secondary metabolites or natural products. These compounds are of interest to humans because they exhibit bioactive structures or substructures in various organisms, including humans [2].

Secondary metabolites, although not essential for survival, play a crucial role in the organism’s adaptation to various phenomena. The production of these metabolites may be activated at specific developmental stages, as well as in stressful situations such as nutritional deficiencies, hostile environments, or pathogen attacks.

**Figure 1:**
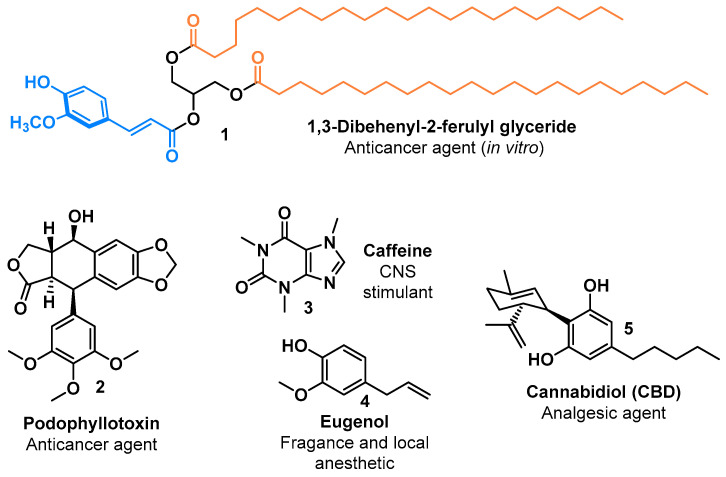
Representative natural products with significant biological and recreational activity.

Throughout human history, secondary metabolites have been used to improve the quality of life in various aspects, ranging from the food industry to the clinical setting for the treatment of diverse diseases. For example, podophyllotoxin (**2**) is used as an anticancer agent; caffeine (**3**) is known for its ability to stimulate the central nervous system (Figure 1); cinnamaldehyde, linalool, and eugenol (**4**) are compounds used in perfumes and essences; and cannabidiol (**5**), the active component of marijuana, has been approved as an analgesic and as an adjunct to reduce the effects of chemotherapy [3,4,5,6].

A specific example is the natural product **1**, which was isolated from *Aquilaria malaccensis*; this genus comprises tropical evergreen trees, which are native to and cultivated in Asia, mainly in the Southeast region. This genus is very popular because some of its species produce a substance known as agarwood. Agarwood began to be used in a wide variety of treatments within traditional Asian medicine as a remedy for colds, vomiting, gastric pain, chest pain, abdominal pain, and many other ailments [7].

This substance (agarwood) has multiple uses in traditional Asian medicine and in industries, such as cosmetics. Agarwood has been evaluated on numerous occasions to elucidate its components and identify potential natural products; it remains highly popular due to its unique aroma, making it a luxury material in industries such as perfumery and cosmetics. However, its high demand and rarity in nature have led to the overexploitation of the producing species to the point that several *Aquilaria* species have been classified as “vulnerable” and “endangered.” Consequently, as indicated by Li et al. in 2023, these species are under strict monitoring by the Convention on International Trade in Endangered Species of Wild Fauna and Flora (CITES) [8].

In an analysis carried out by Gunasekera and collaborators in 1981 [9], the natural product **1** showed in vitro anticancer activity against P-388 lymphocytic leukemia, and was identified which has a triacylglycerol (TAG) structure. TAGs consist of three fatty acids attached via ester bonds to a single glycerol molecule. They are generally classified as simple—when the same fatty acid occupies all three positions—or mixed, when two or three different fatty acids are present in their structure [10].

In compound **1**, positions 1 and 3 of the main chain are replaced by docosanoic acid (commonly known as behenic acid), a 22-carbon fatty acid. Meanwhile, at position 2 of the main chain, there is a bond to a molecule of ferulic acid, which belongs to the group of molecules known as hydroxycinnamic acids (HCAs). HCAs are a group that has been widely studied for their pharmacological properties, mainly for their antioxidant and photoprotective effects.

**Scheme 1:**
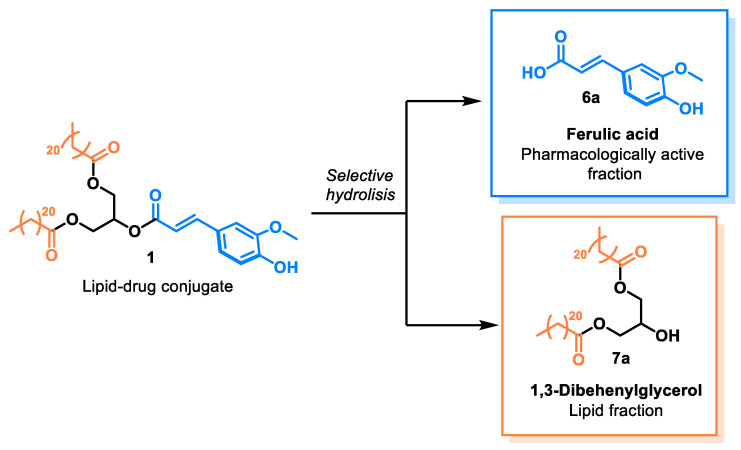
Natural product 1,3-dibehenyl-2-ferulyl glyceride (**1**) and the components from which it is derived: the pharmacologically active fraction and the lipid fraction.

Within the structure of the natural product 1,3-dibehenyl-2-ferulyl glyceride (**1**), two fractions can be identified: a pharmacologically active fraction (in this case, the ferulic acid molecule) and a lipid fraction corresponding to the remainder of the molecule (in this case, a 1,3-diacylglycerol fraction). Chemical entities that have these fractions linked together are known as lipid prodrugs or lipid–drug conjugates (Scheme 1).

According to the Scifinder database, the total synthesis of the natural product **1** has not yet been reported. The synthetic production of 1,3-dibehenyl-2-ferulyl glyceride would allow for the evaluation of its biological activity as an antioxidant or as a photoprotective component for future commercial products. However, given the low availability of natural product **1** at the time of extraction, coupled with the endangered status of the *Aquilaria malaccensis* plant, it is convenient to develop an optimal and suitable synthetic route that allows obtaining the desired natural product and, subsequently, carrying out evaluations on its biological effects [11].

**Scheme 2:**
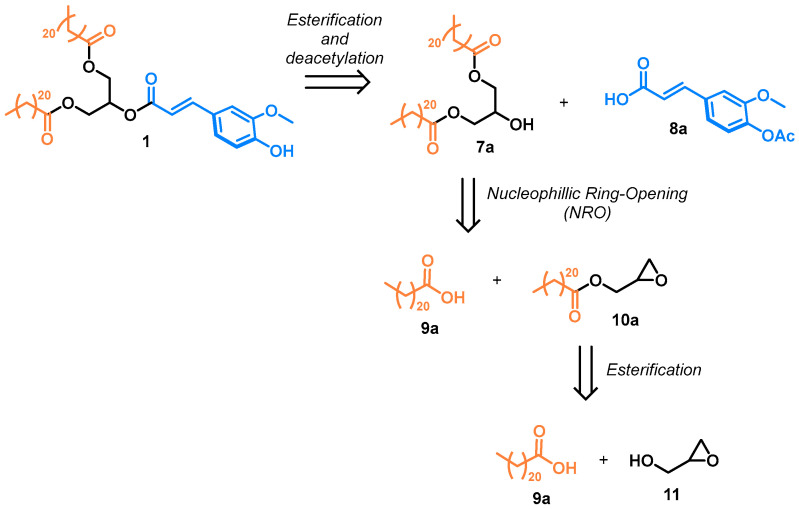
Retrosynthetic analysis for preparation of compound **1**.

The retrosynthetic analysis was carried out to prepare compound **1**, which can be obtained via esterification of 1,3-diacylglycerol **7a** with *O*-acetylated ferulic acid (**8a**), followed by deacetylation, as shown in Scheme 2. Diacylglycerol **7a** would be obtained through the nucleophilic ring-opening reaction between behenic acid (**9a**) and glycidyl behenate (**10a**), based on a previously reported methodology [12]. Finally, glycidyl behenate (**10a**) could be accessed via esterification of behenic acid (**9a**) with glycidol (**11**), using the Mitsunobu reaction due to the lability of **10a** to acid catalysis.

## 2. Results and Discussion

**Scheme 3:**
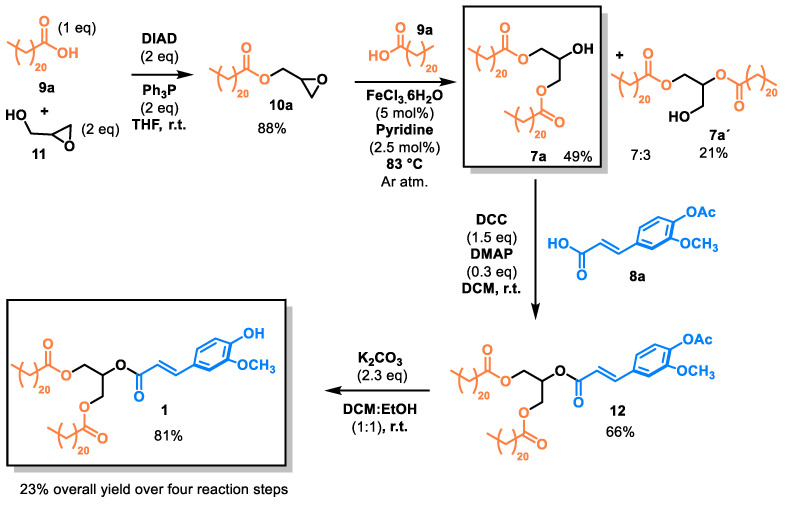
Synthetic route for obtaining the natural product 1,3-dibehenyl-2-feruryl glyceride (**1**).

The synthetic route to product **1** consisted of four steps (Scheme 3), but problems emerged in the initial proposed route as the project progressed. This section describes each reaction performed and explains the adjustments made to the route. For the formation of glycidyl behenate (**10a**), the Mitsunobu reaction between behenic acid (**9a**) and an excess of glycidol (**11**) was carried out, affording the product in good yield. Subsequently, glycidyl behenate (**10a**) was reacted with behenic acid (**9a**) in the presence of iron(III) chloride and pyridine to obtain a mixture of 1,3-dibehenyl glycerol (**7a**) and 1,2-dibehenyl glycerol (**7a′**), which was separable by column chromatography. Product **12** was formed via a Steglich esterification by condensing 1,3-dibehenyl glycerol (**7a**) with *O*-acetylated ferulic acid (**8a**). Finally, deacetylation of compound **12** under weakly basic conditions afforded the natural product **1** in good yield.

**Figure 2:**
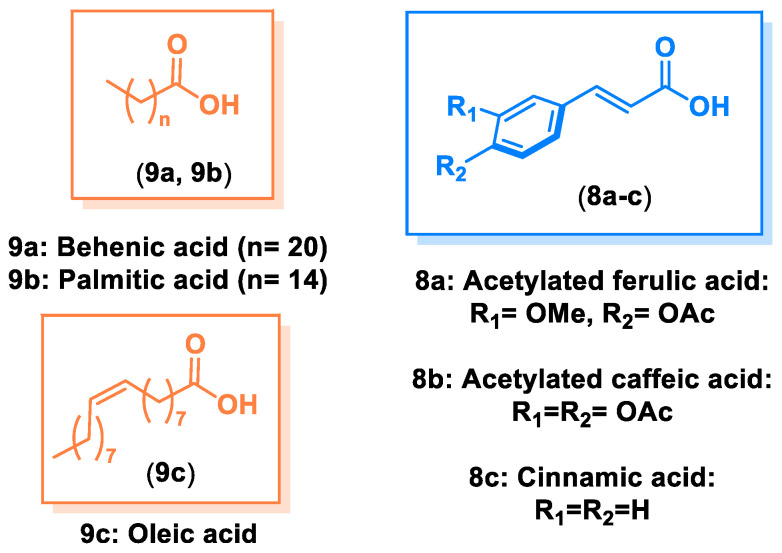
Chemical structures of the fatty acids (**9a**–**c**) and cinnamic acid derivatives (**8a**–**c**) used in this work.

Further derivatives of product **1** were prepared by varying the fatty acid at positions 1 and 3 of the triacylglycerol. The natural product contained behenic acid (docosanoic acid, **9a**; a C22 saturated fatty acid). Palmitic (**9b**; C16 saturated) and oleic acids (**9c**; C18 monounsaturated, Δ9) were also employed (Figure 2). At position 2 of the 1,3-diacylglycerol, coupling was performed with two additional compounds: acetylated caffeic acid (**8b**) and cinnamic acid (**8c**) (Figure 2).

Notably, palmitic acid was used to optimize each of the reaction steps described herein, owing to its low cost and laboratory availability.

### 2.1. Obtaining Glycidyl Esters

The main chemical alternative for achieving ester bond formation involves generating more reactive species through the activation of the functional groups involved: carboxylic acids and alcohols. In general, the reaction mechanisms are based on the direct or indirect dehydration of carboxylic acids [13]. Perhaps the best-known method for ester synthesis is the Fischer esterification reaction [14], notable for its simplicity and practicality [15]. Its main drawback, however, is the equilibrium established between reactants and products at each step of the process. This limitation can be mitigated by selecting appropriate conditions that shift the equilibrium toward the products.

Two main strategies, which can also be combined, are commonly employed: (1) Use of a large excess of the nucleophile (alcohol), ideally as the solvent. (2) Removal of the byproduct (water) as it forms, thereby suppressing the reverse reaction [16].

Nevertheless, the presence of equilibrium and the requirement for acid catalysis considerably restrict the applicability of the Fischer reaction in many synthetic strategies. Consequently, alternative methodologies have been developed to facilitate ester formation, such as the Mitsunobu reaction and Steglich esterification.

Since its discovery, the Mitsunobu reaction has been a cornerstone of synthetic chemistry [17]. Its importance grew considerably from the 1990s onward, driven by its applications in the synthesis of natural products and their synthetic derivatives. Since then, continuous efforts have been made to render the reaction catalytic through the development of new reagents. The Mitsunobu reaction involves the coupling of a primary or secondary alcohol with a pronucleophile (NuH) in the presence of an azodialkyl dicarboxylate and a trialkyl- or triarylphosphine. It proceeds under mild conditions at neutral pH, with reaction temperatures ranging between 0 °C and room temperature. This process is classified as a dehydrative coupling. Oyo Mitsunobu first described the conversion of primary and secondary alcohols into esters using phosphines and azodicarboxylates—specifically DEAD or DIAD—as mediating reagents [18]. Initially, this transformation represented a novel route to esters that are typically difficult to obtain through conventional esterification methods. However, the full potential of the reaction was unleashed upon the discovery that it also enables the formation of carbon–heteroatom (O, N, S) bonds [19]. The reaction mechanism has been extensively studied using various experimental and theoretical approaches and is now considered to be well understood [18,20].

Our synthetic route began with the coupling of fatty acids **9a**–**c** with glycidol (**11**) via ester bond formation using diisopropyl azodicarboxylate (DIAD) and triphenylphosphine (Ph_3_P) as acyl migration reagents under Mitsunobu-type conditions, affording the corresponding glycidyl esters. The reaction was carried out at room temperature for 12 h, as summarized in Table 1. This transformation does not present major drawbacks; therefore, the optimization efforts focused on improving the yield by varying the solvent and the equivalents of DIAD, Ph_3_P, and glycidol. Under optimized conditions, good yields between 80% and 88% were achieved (experiments 4–8, Table 1), depending on the fatty acid used. Two equivalents of glycidol, along with DIAD and PPh_3_, were required to obtain higher yields. The reaction proceeded well in both dichloromethane (DCM) and tetrahydrofuran (THF); however, the latter offered the advantage that the byproducts—triphenylphosphine oxide and reduced DIAD—could be removed by precipitation via selective crystallization after standing overnight in a refrigerator.

The idea of forming glycidyl esters arose from reviewing various articles on the synthesis of monoacylglycerols (MAG), as these esters are known precursors for the synthesis of diacylglycerols (DAG) [12,21].

### 2.2. Epoxide NRO. Synthesis of 1,3-Diacylglycerols (1,3-DAG)

Once the corresponding glycidyl esters **10a**–**c** were obtained, the synthesis of 1,3-diacylglycerols (1,3-DAG) **7a**–**c** was pursued via nucleophilic ring opening (NRO) of the epoxide. Initially, NRO was carried out using phase-transfer catalysts, specifically benzyltriethylammonium chloride (BTEAC) and tetrabutylammonium bromide (TBAB), in catalytic amounts (4 mol%) under solvent-free conditions at various temperatures, following previously reported procedures [12,21].

**Scheme 4:**
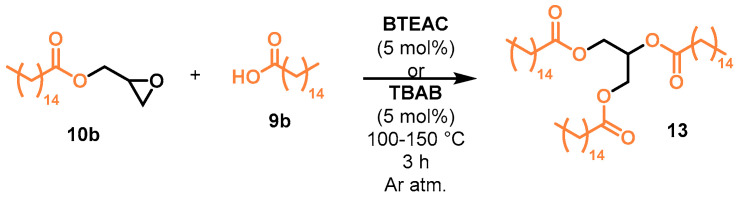
Undesired synthesis of glycerol tripalmitate **13**.

The first tests were conducted using 4 mol% BTEAC at 150 °C for 3 h. However, under these conditions, tripalmitoylglycerol (**13**) was obtained as shown in experiment 1, Table 2. Subsequent tests were performed by replacing the phase-transfer catalyst with TBAB and lowering the reaction temperature to 100 °C (experiment 2, Table 2). After approximately 3 h, the product obtained was again the compound **13**, though in an undetermined yield (Scheme 4).

**Scheme 5:**
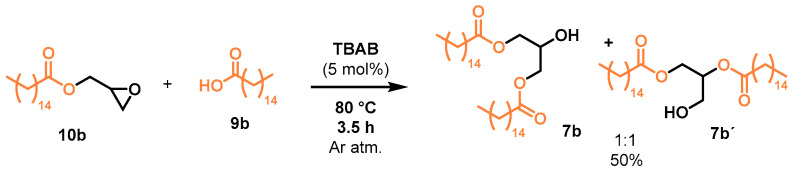
Synthesis of glycerol dipalmitates **7b** and **7b’**.

To avoid the formation of the triester **13**, the temperature was reduced to below 100 °C. For instance, in the preparation of 1,3-dipalmitoylglycerol **7b**, a temperature of 80 °C was used, which allowed both palmitic acid **9b** and glycidyl palmitate **10b**—both solids at room temperature—to melt. However, the main drawback of this method was its low regioselectivity, as it produced both the 1,3-dipalmitoylglycerol (**7b**) and the 1,2-dipalmitoylglycerol (**7b′**) isomers in a 1:1 ratio (experiment 3, Table 2), with a combined yield of 50% (Scheme 5).

Therefore, alternative methods were explored to improve both the yield and the regioselectivity of the reaction.

Zhao Y. and colleagues [22] developed a method for achieving NRO through dual activation between iron(III) chloride (FeCl_3_) and pyridine (Py). An advantage of this method is that it is solvent-free; therefore, appropriate modifications were made to our protocol to maintain this characteristic. The protocol was based on the combined melting of **9a**–**c** and **10a**–**c**, corresponding to the same ester chain, with catalytic amounts of iron(III) chloride hexahydrate (5 mol%) and pyridine (2.5 mol%). The mixture was heated to the melting point of the fatty acids; for palmitic acid **9b**, the temperature was around 65 °C, while for behenic acid **9a**, it was approximately 83 °C. In the case of glycidyl oleate **10c** and oleic acid **9c**, both reagents were in a liquid state, but they were still heated to approximately 60 °C to obtain better yields. All tests in this reaction step were carried out under an inert argon atmosphere (Table 2).

Several trials were conducted in which the concentration of both catalysts was modified, using catalyst loadings of 2.5–5 mol% (experiments 4–9, Table 2). Trials were also performed using only one catalyst. When iron(III) chloride hexahydrate and pyridine were employed, the desired isomer **7b** was obtained with slightly higher selectivity for the major isomer and a combined yield of 50% (experiment 4, Table 2). Interestingly, when only iron(III) chloride hexahydrate was used as the catalyst, the undesired isomer **7b′** was obtained as the major product in moderate yield (experiment 5, Table 2). When pyridine was used alone, a complex mixture formed, and the products could not be isolated (experiment 6, Table 2). The best outcome was obtained employing iron(III) chloride hexahydrate (5 mol%) in combination with pyridine (2.5 mol%), affording an increased yield of 70% with appreciable selectivity toward the desired product **7b** (experiment 7, Table 2).

The conditions shown in Table 2 were chosen because they yielded the highest results: 71% for 1,3-dipalmitate (**7b**), 62% for 1,3-dioleate (**7c**), and 59% for 1,3-dibehenate (**7a**). Higher regioselectivity for these examples was also observed, with a 7:3 ratio for the desired 1,3-DAG isomers **7a**–**c** (experiments 7–9, Table 2).

### 2.3. Esterification of 1,3-DAG with HCA

This step represented the greatest challenge during the synthesis process. The coupling was carried out under mild conditions because our precursors **7a**–**c** (1,3-DAG) are sensitive to hydrolysis under classical Fischer esterification conditions; therefore, different esterification techniques were attempted.

The reaction between **7b** and ferulic acid (**6a**) was attempted under Mitsunobu-type and Steglich-type conditions. After several attempts without obtaining the esterified product, it was concluded that the phenolic hydroxyl groups might be interfering with product formation. Due to the high steric hindrance of our 1,3-DAG, condensation between the carboxylate and phenol moieties of two different ferulic acid molecules may have occurred [20]. To overcome this issue, the phenolic hydroxyl groups of ferulic and caffeic acids were protected via an acetylation reaction using a slight excess of acetic anhydride as the acetyl group source and pyridine as the solvent, following a reported procedure [23].

Subsequently, the coupling was carried out under the two aforementioned conditions, and the desired product was obtained via a Steglich-type reaction [24]. This reaction was performed by dissolving the corresponding 1,3-DAG (**7a**–**c**), HCA (**8a**–**c**), and DMAP in anhydrous DCM. Once dissolved, the mixture was cooled to 0 °C for the addition of DCC until complete solubilization. The reaction was maintained at 0 °C for 30 min and then allowed to warm to room temperature for a total of 48 h, yielding the desired derivatives of the acetylated natural product in moderate to good yields, as shown in Table 3. When using the diacetylated derivatives of caffeic acid (experiments 2, 5, and 8), the yields of the caffeic acid ester were low (26–34%), likely because in a basic medium (due to the presence of DMAP), the diacetylated caffeic acid moiety is more susceptible to deprotection under prolonged reaction times.

In the case of the tests performed under Mitsunobu-type conditions, the desired coupling was not achieved. After analyzing the reaction mechanism and its limitations, a possible explanation for the inefficiency of this reaction compared to Steglich-type esterification was found. Both Steglich and Mitsunobu reactions require deprotonation of the carboxylic acid in question; however, in the Mitsunobu-type reaction, it acts as a nucleophile, while in the Steglich reaction, it has a dual nucleophile–electrophile role.

Once the carboxylic acid is deprotonated, both reactions aim to activate a reagent, and this is possibly where the success of the Steglich reaction lies. In the Mitsunobu reaction, after deprotonation of the carboxylic acid and formation of the betaine-type intermediate **24**, a nucleophilic attack by the alcohol **7** on this intermediate is assumed. Under our conditions, the steric hindrance of adduct **24**, combined with the steric hindrance of 1,3-DAG **7**, likely prevented the formation of the alkoxyphosphonium salt, and consequently, the reaction stopped at this step.

**Scheme 6:**
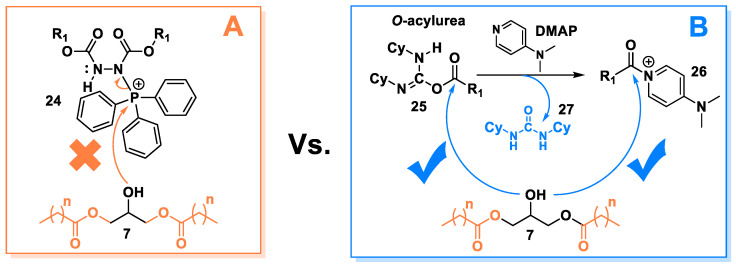
Comparison between esterification methods of (**A**): Mitsunobu reaction vs. (**B**): Steglich reaction, with 1,3-DAG used in this work. Cy = Cyclohexyl.

In comparison, in the Steglich reaction, once the carboxylate is formed, it attacks DCC to form *O*-acylurea **25**. Subsequently, this adduct is attacked by DMAP, forming a new adduct **26** between the carbonyl group and DMAP. This adduct allows for high activation of the carbonyl carbon and facilitates the nucleophilic attack of 1,3-DAG **7** to form the product. In this case, the steric hindrance caused by the betaine and alcohol in the Mitsunobu reaction is eliminated, as shown in Scheme 6.

### 2.4. Deprotection of Phenolic Hydroxyls

The final step in the route was the deacetylation of the phenolic hydroxyls. Deprotection is carried out under basic conditions by reacting the acetylated products with potassium carbonate (K_2_CO_3_) in slight excess (2.3 eq.) dissolved in a methanol/DCM (1:1) mixture at room temperature. The reaction is completed in approximately two hours with good yields (>83%), as shown in Table 4.

The variable considered in this reaction was time. The reaction was monitored by TLC every 10 min until a product more polar than the acetylated products was observed. Close monitoring of the reaction by TLC was very important since these molecules are susceptible to hydrolysis in other regions containing the ester group. The yields of the acetylation are good at short reaction times of 2 to 3 h (>83%, experiments 1 and 2, Table 4).

**Scheme 7:**
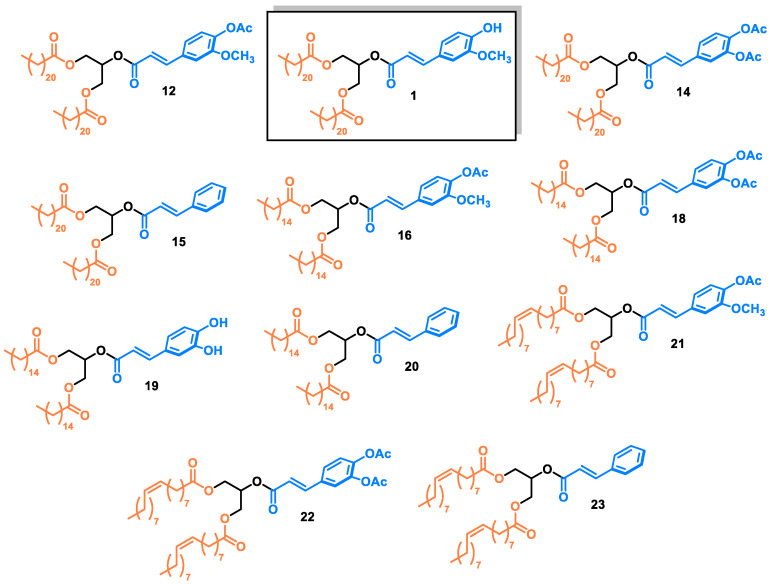
1,3-dibehenyl-2-ferulyl glyceride (**1**) and its derivatives synthesized in this work.

Through this reaction, the desired compound **1** was synthesized for the first time (Scheme 7). The spectroscopic data of the synthetic material were identical to those reported for the natural product. Regarding **1**, in the respective spectroscopic summary (Figures S51–S53 and Table S54 of the supporting information), it is established that a yield of 86% was obtained in this step. The compound **1** showed the expected peaks in the ^13^C and ^1^H NMR spectra, in addition to the signal *m*/*z*: 914 corresponding to protonated molecular ion [M+H]^+^ in LRMS (DART+) (S53). The results of the elemental analysis were C[%] = 75.15 and H[%] = 11.06, which are in good agreement with the theoretical values (C[%] = 74.95 and H[%] = 11.04). The corresponding error percentages are less than 0.4%, thus confirming the identity of the natural product. All the final products are shown in Scheme 7.

### 2.5. Biological Activity

Currently, the antioxidant capacity activity tests were obtained using the 2,2-diphenyl-1-picrylhydrazyl (DPPH) radical scavenging test for the compounds **14**, **15**, and **18**–**23**. Among them, compound **19** was found to have interesting antioxidant activity. Then, a comparative study was carried out to measure the antioxidant capacity between 1,3-dipalmityl-2-caffeoyl glyceride (**19**) and two compounds used as standard references, trolox (6-hydroxy-2,5,7,8-tetramethylchroman-2-carboxylic acid) and gallic acid (3,4,5-trihydroxybenzoic acid). The result of this study was obtaining the IC_50_ value of the tested compound.

Table 5 presents the DPPH radical scavenging activity report. Compound **19** exhibits higher antioxidant activity than trolox but lower than gallic acid. Although compound 19 is a molecule that triples the molecular weight of trolox and quadruples that of gallic acid, its antioxidant capacity is due to the presence of a 1,2-catechol group, known for scavenging free radicals and protecting against oxidative stress in biological and food systems [25]. Its catechol structure is essential for donating hydrogen atoms and would have the ability to chelate metals.

## 3. Materials and Methods

All reagents and solvents were obtained from Sigma-Aldrich (St. Louis, MO, USA) and Fluka (Seelze, Germany). THF was distilled in the presence of sodium/benzophenone, and hexane and dichloromethane were distilled in the presence of calcium hydride prior to use. Melting points were determined using a Fisher–Johns (Fisher Scientific, Waltham, MA, USA) apparatus and are uncorrected. All reactions were carried out under an argon atmosphere unless otherwise specified. Reaction progress was monitored by thin-layer chromatography using silica GF plates obtained from Merck (Darmstadt, Germany). The plates were visualized using a short-wave UV lamp (254 nm) model UVP UVG-11 (Jena, Germany) and/or with a vanillin, phosphomolybdic acid, or potassium permanganate developer. ^1^H and ^13^C NMR spectra were run using Jeol Eclipse-300 MHz (Tokyo, Japan) and Bruker Avance III-400 MHz instruments (Billerica, MA, USA) with CDCl_3_ as the solvent. Chemical shifts are reported in parts per million (ppm) using tetramethylsilane as a reference (δ = 0.0 for 1H) or by the CDCl_3_ signal. Coupling constants are reported in hertz (Hz). IR spectra were obtained using a Bruker Tensor 27 FT-IR instrument (USA). Low-resolution mass spectra using the DART+ technique were obtained on a Jeol JMS-T100LC spectrometer (Japan).

All products reported here were obtained in a similar manner. Although the procedure for obtaining one of them (1,3-Dibehenyl-2-ferulyl glyceride, **1**) is specified in this section, if further information is required, please revise the Supporting Information file.

Example of the preparation of compound **1**: In a 50 mL round-bottom flask, 0.030 g of 1,3-Dibehenyl-2-(4-acetyl)ferulylglyceride (**12**) (0.032 mmol) was added. The reagent was dissolved in 7 mL of a 1:1 DCM/methanol mixture. Subsequently, 0.014 g of potassium carbonate (0.100 mmol) was added. The reaction was stopped after 3 h. Purification was performed by column chromatography using an 8:2 hexane/ethyl acetate system. A whitish amorphous solid was obtained in an amount of 0.024 g, with an 86% yield. A whitish amorphous solid was obtained in an amount of 0.024 g, with an 86% yield. For further information, the spectroscopic summary of this and other compounds in the work can be found in the article’s Supporting Information file.

The measurement of DPPH radical activity was carried out under the following experimental conditions: Temperature 37 ± 2 °C; incubation time 30 ± 2 min; DPPH concentration 100 µM; the experiment carried out to generate the report was a curve with a DMSO vehicle.

## 4. Conclusions

The efficient synthesis of 1,3-dibenhenyl-2-ferulyl glyceride (**1**) and its derivatives was achieved, with overall yields exceeding 23%. This compound is currently extracted from a natural product of great interest to the cosmetical al industry, owing to its structural properties, making it an excellent candidate for evaluation in key biological tests, in this work, the IC_50_ value was obtained for 1,3-dipalmityl-2-caffeoyl glyceride (**19**), with good results, leaving as a prospect a more in-depth test for compounds analogous to 1,3-dibenhenyl-2-ferulyl glyceride (**1**), including the determination of the IC_50_ value. The products obtained at each step of the proposed process were also characterized using common spectroscopic techniques, leading to their individual characterization.

## Figures and Tables

**Table 1:** Optimization of the glycidyl esters synthesis.

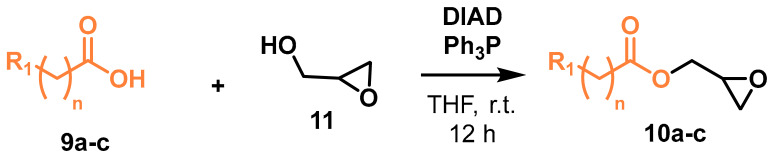					
Experiment	Fatty Acid	Solvent	Glycidol Equivalents	Ph_3_P and DIAD Equivalents	Yield (%)
1	Palmitic acid **9b**	DCM	1.0	1.0	55
2	Palmitic acid **9b**	DCM	1.0	1.0	60
3	Palmitic acid **9b**	DCM	1.2	1.2	72
4	Palmitic acid **9b**	DCM	1.6	1.6	80
5	Oleic acid **9c**	THF	1.6	1.6	83
6	Behenic acid **9a**	THF	1.6	1.6	76
7	Behenic acid **9a**	THF	2.0	2.0	88

**Table 2:** Optimization of 1,3-diglycerides synthesis.

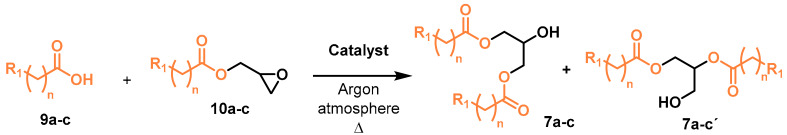						
Experiment	Catalyst (mol %)	Products	Regio- Selectivity (7:7’)	Temperature (°C)	Time (h)	Yield (%)
1	BTEAC (4.0)	TAG **13**	ND	150	2	ND
2	TBAB (4.0)	TAG **13**	ND	100	3	ND
3	TBAB (4.0)	**7b** and **7b’**	1:1	80	3.5	50
4	FeCl_3_•6H_2_O (2.5) Py (2.5)	**7b** and **7b’**	6:4	60–85	24	50
5	FeCl_3_•6H_2_O (2.5)	**7b** and **7b’**	1:9	70	24	50
6	Py (2.5)	Complex mixture	ND	70	24	ND
7	FeCl_3_•6H_2_O (5.0) Py (2.5)	**7b** and **7b’**	7:3	65	48	71
8	FeCl_3_•6H_2_O (5.0) Py (2.5)	**7a** and **7a’**	7:3	83	48	59
9	FeCl_3_•6H_2_O (5.0) Py (2.5)	**7c** and **7c’**	68:32	60	48	62

ND = Not determined.

**Table 3:** Yields obtained in the Steglich reaction between **7a**–**c** and **8a**–**c**.

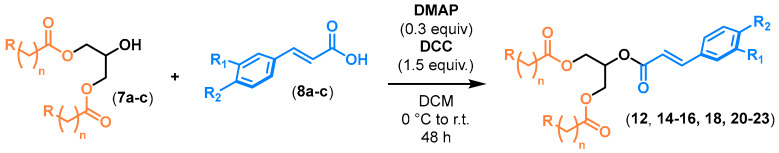				
Experiment	1,3-DAG (7a–c)	HCA (8a–c)	Product (12, 14, 15, 18, 20–23)	Yield (%)
1	**7a**	**8a**	**12**	66
2	**7a**	**8b**	**14**	26
3	**7a**	**8c**	**15**	70
4	**7b**	**8a**	**16**	70
5	**7b**	**8b**	**18**	31
6	**7b**	**8c**	**20**	69
7	**7c**	**8a**	**21**	61
8	**7c**	**8b**	**22**	34
9	**7c**	**8c**	**23**	79

**Table 4:** Yields obtained in the deacetylation for obtaining 1,3-dibehenyl-2-feruryl glyceride (**12**) and analogs (**16** and **18**).

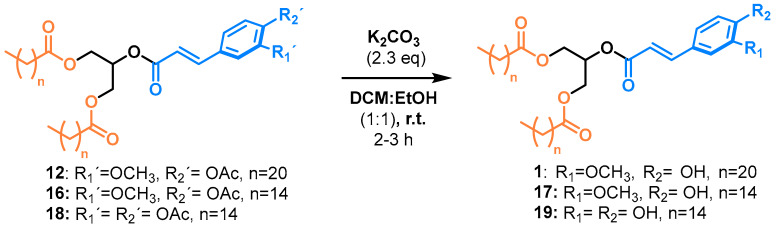						
Experiment	Starting Material	Substituents (R’)	Product	Substituents (R)	Time (h)	Yield (%)
1	**12**	R_1_’ = OCH_3_ R_2_’ = OAc	**1**	R_1_ = OCH_3_ R_2_ = OH	3	86
2	**18**	R_1_’ = R_2_’ = OAc	**19**	R_1_ = R_2_ = OH	2	83

**Table 5:** DPPH radical scavenging activity.

Sample	Concentration	O. D. (515 nm)	DPPH Reduction (%)	IC50
**19**	DPPH	0.608 ± 0.005		17.58 ± 0.45 µM
	5.62 µM	0.512 ± 0.001 **	15.8 ± 0.45 **	
	10 µM	0.431 ± 0.004 **	29.24 ± 0.83 **	
	17.78 µM	0.306 ± 0.005 **	49.72 ± 1.08 **	
	31.62 µM	0.145 ± 0.005 **	76.16 ± 0.91 **	
	56.23 µM	0.100 ± 0.001 **	83.55 ± 0.10 **	
	100 µM	0.088 ± 0.001 **	85.59 ± 0.12 **	
Trolox	DPPH	0.640 ± 0.006		19.57 ± 0.31 µM
	3.16 µM	0.590 ± 0.006 **	7.88 ± 0.34 **	
	5.62 µM	0.549 ± 0.008 **	14.19 ± 0.57 **	
	10 µM	0.479 ± 0.008 **	25.13 ± 0.53 **	
	17.78 µM	0.359 ± 0.009 **	43.90 ± 0.84 **	
	31.62 µM	0.144 ± 0.010 **	77.59 ± 1.26 **	
	56.23 µM	0.028 ± 0.001 **	95.59 ± 0.14 **	
	100 µM	0.026 ± 0.001 **	96.02 ± 0.11 **	
Gallic acid	DPPH	0.640 ± 0.006		8.76 ± 0.89 µM
	1 µM	0.584 ± 0.003	8.67 ± 0.88 **	
	3.16 µM	0.507 ± 0.007 **	20.78 ± 1.51 **	
	10 µM	0.278 ± 0.035 **	56.52 ± 5.81 **	
	31.62 µM	0.034 ± 0.000 **	94.62 ± 0.07 **	
	100 µM	0.034 ± 0.000 **	94.74 ± 0.09 **	

Values represent the mean of three independent experiments (*n* = 3) ± standard error of the mean. Data were subjected to an analysis of variance (ANOVA) followed by a Dunnett’s test to isolate groups with significant differences. Values of *p* ≤ 0.01 (**) were considered significant differences with respect to DPPH.

## Data Availability

The original contributions presented in this study are included in the article/Supplementary Materials. Further inquiries can be directed to the corresponding authors.

## Supplementary Materials

The following supporting information can be downloaded at: https://www.mdpi.com/article/10.3390/molecules31101593/s1, Synthesis and characterization of the products **7a**–**c**, **10a**–**c**, **16**, **18**, **20**–**23**, **12**, **14**–**15**, **19**, and **1** are in S1–S17, Figures S18–S50 contain the Spectra (^1^H NMR and ^13^C NMR) of the products **7a**–**c**, **10a**–**c**, **16**, **18**, **20**–**23**, **12**, **14**–**15**, **19**, and finally Figures S51–S53 and Table S54 contain the Spectra (^1^H NMR, ^13^C NMR, and MS), and the elemental analysis report with percentage of error obtained for the product **1** (1,3-dibehenyl-2-feruryl glyceride).

## Abbreviations

The following abbreviations are used in this manuscript:

1,3-DAG	1,3-Diacylglycerol or 1,3-diglycerides
^1^H NMR	Nuclear Magnetic Resonance of Hydrogen 1
^13^C NMR	Nuclear Magnetic Resonance of Carbon 13
BTEAC	Benzyltriethylammonium chloride
CDCl_3_	Deuterated chloroform
Cy	Cyclohexyl
DCC	N,N′-Dicyclohexylcarbodiimide
DCM	Dichloromethane
dd	Double of doubles
DIAD	Diisopropyl azodicarboxylate
DMAP	4-(Dimethylamino)pyridine
HCA	Hydroxicinnamic acids
MHz	Megahertz
nm	Nanometers (1 nm = 10^−9^ m)
NRO	Nucleophilic Ring Opening
Ph_3_P	Triphenylphosphine
ppm	parts per million
Py	Pyridine
r.t.	Room temperature
TAG	Triacylglycerol
TBAB	Tetrabutylammonium bromide
THF	Tetrahydrofurane
UV	Ultraviolet region of the electromagnetic spectrum
