# Supplementary material for: Synthesis of the Natural Product 1,3-Dibehenyl-2-ferulyl Glyceride (Aquilaria malaccensis) and Its Derivatives
Source: Molecules. 2026 May 9;31(10):1593. doi: 10.3390/molecules31101593 (PMC13209407; doi:10.3390/molecules31101593)
Supplement: Supplementary file 1 [file molecules-31-01593-s001.zip › molecules-4204437-supplementary.pdf]

Supporting information

Synthesis of the Natural Product 1,3-Dibehenyl-2-ferulyl Glyceride (Aquilaria malaccensis) and Its Derivatives

Alexis de Jesús Sánchez-Esparza [1], Margarita Cantú-Reyes [2], Amira Jalil Fragoso-Medina [3], Antonio Nieto-Camacho [1], Miguel Ángel Ortega-Ruiz [1,2], Oscar Yael Osorio-Godínez [1,2], Alejandra Chávez-Riveros [2], Gabriel Cuevas[1] * and David Atahualpa Contreras-Cruz [1,2] *.

1 Instituto de Química, Universidad Nacional Autónoma de México, Ciudad de México. C.P. 04510. alexis.qfb.sanchezesparza@gmail.com (A.J. S.-E.)
2 Facultad de Estudios Superiores Zaragoza, Universidad Nacional Autónoma de México, Ciudad de México. C.P. 09230, México; cantureyes.margarita@zaragoza.unam.mx (M.C.-R.)
3 Instituto de Física, Universidad Nacional Autónoma de México, Ciudad de México. C.P. 04510, México; amira@fisica.unam.mx
* Correspondence: gecgb@unam.mx (G.C.); atahualpa_c@zaragoza.unam.mx (D.A.C.-C.); Tel.: 55 7365 8936.

Table of contents

1. Synthesis and characterization of the products **7a-c**; **10a-c**; **16, 18, 20-23, 12, 14-15, 19,** and **1**.	S1
2. Spectra(^{1}H NMR and ^{13}C NMR) of the products **7a-c**; **10a-c**; **16, 18, 20-23, 12, 14-15**, and **19**.	S18
1,3-dibehenyl glycerol (**7a**); S18-S19	
1,3-Dipalmityl glycerol (**7b**); S20-21	
1,3-Dioleyl glycerol (**7c**); S22-S23	
Glycidyl behenate (**10a**); S24-S25	
Glycidyl palmitate (**10b**); S26-S27	
Glycidyl oleate (**10c**); S28-S29	
1,3-Dipalmityl-2-(4-acetyl)ferulyl glyceride (**16**); S30-S31	
1,3-Dipalmityl-2-(3,4-diacetyl)caffeoyl glyceride (**18**); S32-S33	
1,3-Dipalmityl-2-cinnamyl glyceride (**20**); S34-S35	
1,3-Dioleoyl-2-(4-acetyl)ferulyl glyceride (**21**);S36-S37	
1,3-Dioleoyl-2-(3,4-diacetyl)caffeoyl glyceride (**22**); S38-S39	
1,3-Dioleoyl-2-cinnamyl glyceride (**23**); S40-S41	
1,3-Dibehenyl-2-(4-acetyl)ferulyl glyceride (**12**); S42-S44	
1,3-Dibehenyl-2-(3,4-diacetyl)caffeoyl glyceride (**14**); S45-S46	
1,3-Dibehenyl-2-cinnamyl glyceride (**15**); S47-S48	
1,3-Dipalmityl-2-caffeoyl glyceride (**19**); S49-S50	
3. Analyses of the product **1** (1,3-dibehenyl-2-feruryl glyceride)	S51

	Spectra (^{1}H NMR, ^{13}C NMR, and MS); S51-S53
	Elemental analysis report and percentage of error obtained; S54

1. Synthesis and characterization of the products 7a-c; 10a-c; 16, 18, 20, 21, 22, 23, 12, 14,15, 17, 19, and 1. S1-S17.

S1. Synthesis of 1,3-Dibehenate (7a): In a 100 mL round-bottom flask, 0.94 g of 95% behenic acid (2.64 mmol) was added. The reaction was performed under an inert atmosphere by supplying argon with a balloon and heated until complete melting of the reagent was achieved (80°C). Subsequently, 0.011 g (0.063 mmol) of iron (III) chloride hexahydrate and 1.05 µL of pyridine were added, and stirring was maintained for a couple of minutes. Finally, 1.00 g of glycidyl behenate (**10a**) (2.52 mmol) was added, while maintaining heating and the inert atmosphere. The reaction was kept heated for 48 hours. The reaction mixture was purified by column chromatography using a 90:6:4 system (Hexane: DCM: Ethyl acetate). Three fractions were obtained; In the first fraction, 0.59 g of product **7a** was obtained, in the second fraction, 0.33 g of a mixture of products **7a - 7a′** (1,2-Dibehenate) was obtained, and in the last fraction, 0.13 g, which was mainly composed of 7**a′**. A yield of 59% was obtained with a 7:3 ratio of **7a:7a′** isomers.
Spectroscopic summary: ^{1}H NMR ($CDCl_3$, 400 MHz, 25 °C) δ = 0.87 (t, *J* =7.0 Hz, 6H, 2CH_3), 1.21 – 1.34 (m, 72H, 36CH_2), 1.63 (t, *J* = 7.3 Hz, 4H, 2CH_2C**H₂**CH_2OCO), 2.34 (t, *J* = 7.6 Hz, 4H, 2C**H₂**OCO), 4.06 – 4.10 (m, 1H, CH_2C**H**OHCH_2), 4.13 (dd, *J* = 11.4, 5.6 Hz, 2H, C**H₂**CHOH), 4.19 (dd, *J* = 11.3, 4.3 Hz, 2H, C**H₂**CHOH). ^{13}C NMR ($CDCl_3$,100 MHz, 25°C) δ = 14.2, 22.8, 25.0, 29.3, 29.4, 29.5, 29.6, 29.8-29.9, 32.1, 34.3, 65.2, 68.6, 174.1; LRMS (DART+) *m/z*: 720 $[M - OH]^+$.

S2. Synthesis of 1,3-Dipalmitate (7b): A procedure similar to that used for product **7a** was followed. In a 100 mL round-bottom flask, 0.88 g of 98% palmitic acid (**9b**) (3.36 mmol) was added. The reaction was performed under an inert atmosphere by supplying argon with a balloon and heated until the reagent was completely melted (70°C). Subsequently, 0.014 g (0.080 mmol) of iron(III) chloride hexahydrate and 1.30 µL of pyridine (0.001 mmol) were added, and stirring was maintained for a couple of minutes. Finally, 1.00 g of glycidyl palmitate (**10b**) (3.2 mmol) was added, while maintaining heating and the inert atmosphere. The reaction was heated for 48 hours. The reaction mixture was purified by column chromatography using an 8:2:1 system (Hexane: DCM: Ethyl acetate). Three fractions were obtained; In the first fraction, 0.3299 g of product **7b** was obtained, in the second fraction, 0.2180 g of a mixture of products **7b - 7b′** (1,2-Dibehenate) was obtained, and in the last fraction, 0.1083 g, which was mainly composed of **7b′,** was obtained. A yield of 71% was obtained with a ratio of 67:33 of isomers **7b: 7b′**.
Spectroscopic summary: ^{1}H NMR ($CDCl_3$, 400 MHz, 25 °C) δ = 0.87 (t, *J* =7.0 Hz, 6H, 2CH_3), 1.25 (s, 48H, 24CH_2), 1.63 (p, *J* = 7.3 Hz, 4H, 2 CH_2C**H₂**CH_2OCO), 2.34 (t, *J* = 7.6 Hz, 4H, 2 C**H₂**OCO), 4.06 – 4.10 (m, 1H, CH_2C**H**OHCH_2), 4.13 (dd, *J* = 11.4, 5.6 Hz, 2H, C**H₂**CHOH), 4.19 (dd, *J* = 11.3, 4.3 Hz, 2H, C**H₂**CHOH). ^{13}C NMR ($CDCl_3$,100 MHz, 25°C) δ = 14.3, 22.8, 25.0, 29.3, 29.4, 29.5, 29.6, 29.7-29.8, 32.1, 34.3, 65.2, 68.6, 174.1; LRMS (DART+) *m/z*: 551 $[M-OH]^+$.

S3. Synthesis of 1,3-Dioleate (7c): In a 100 mL round-bottom flask, 0.83 g of oleic acid (**9c**) (2.95 mmol) was added. The reaction was performed under an inert atmosphere by supplying argon with a balloon and heated to 65°C. Subsequently, 0.04 g (0.150 mmol) of iron(III) chloride hexahydrate and 0.012 mL of pyridine were added, and stirring was maintained for a couple of minutes. Finally, 1.00 g of glycidyl oleate (**10c**) (2.95 mmol) was added, while maintaining heating and the inert atmosphere. The reaction was continued under heating for 48 hours. The reaction mixture was purified by column chromatography using the 95:5 system (hexane:ethyl acetate). Three fractions were obtained; In the first fraction, 0.76 g of product **7c** was obtained, in the second fraction, 0.19 g of a mixture of products **7c - 7c´**(1,2-Dioleate) was obtained, and in the last fraction, 0.15 g, which was mainly composed of **7c´**, was obtained. A yield of 62% was obtained with a ratio of 68:32 of **7c:7c´** isomers.
Spectroscopic summary: ^1H NMR ($CDCl_3$, 300 MHz, 25 °C) δ = 0.87 (t, J = 6.6 Hz, 6H, $2CH_3$), 1.21 – 1.36 (m, 40H,$20CH_2$), 1.62 (p, J = 7.1 Hz, 4H, $2CH_2C\mathbf{H_2}CH_2OCO$), 2.00 (q, J = 6.3 Hz, 8H, $C\mathbf{H_2}CH{=}CHC\mathbf{H_2}$), 2.34 (t, J = 7.5 Hz, 4H, $C\mathbf{H_2}OCO$), 4.06 – 4.22 (m, 5H), 5.26 – 5.41 (m, 4H, $C\mathbf{H}{=}C\mathbf{H}$). ^{13}C NMR ($CDCl_3$,75 MHz, 25°C) δ = 14.3, 22.8, 25.0, 27.3, 27.4, 29.2, 29.3, 29.5, 29.7, 29.8, 29.9, 32.0, 34.2, 65.2, 68.5, 129.9, 130.2, 174.1 LRMS (DART+) *m/z*: 604 $[M-OH]^+$.

S4. Synthesis of Glycidyl behenate (10a): A procedure similar to that used for obtaining product **10b** was followed. In a 250 mL round-bottom flask, 5.31 g of 95% behenic acid (**9a**) (15.6 mmol) and 8.18 g of triphenylphosphine (31.2 mmol) were added. Both reagents were dissolved in 50 mL of THF (previously distilled and dried). Subsequently, 1.20 mL of 96% glycidol (**11**) was added to the reaction flask. The reaction was carried out for 19 hours. Once complete, the solvent was evaporated from the reaction mixture under reduced pressure. The product obtained was a yellowish amorphous solid, which was added to a flask containing a 97:3 hexane:ethyl acetate mixture to precipitate as much triphenylphosphine oxide as possible. The reaction mixture was decanted, and the liquid phase was impregnated with silica gel. The solvent was then removed by distillation under reduced pressure. Column chromatography was performed using the 95:5 hexane:ethyl acetate system.
Spectroscopic summary: ^1H NMR ($CDCl_3$, 300 MHz, 25 °C) δ = 0.86 (t, J = 6 Hz, 3H, CH_3), 1.24 (s, 36H, $18CH_2$), 1.62 (t, J = 6 Hz, 2H, CH_2), 2.33 (t, J = 7.5 Hz, 2H, CH_2), 2.63 (dd, J_1 = 6 Hz, J_2 = 3 Hz, 1H, CH), 2.83 (dd, J = 4.5, 3 Hz, *1H, CH),* 3.17 – 3.22 *(m, 1H, CH),* 3.89 (dd, J = 12, 6 Hz, 1H, OCH_2), 4.40 (dd, J = 12, 3 Hz, 1H, OCH_2). ^{13}C NMR ($CDCl_3$, 75 MHz, 25°C) δ = 14.2, 22.8, 24.9, 29.2, 29.4, 29.5, 29.6, 29.7, 29.8, 29.8, 32.0, 34.2, 35.7, 44.8, 49.5, 64.9, 70.4, 173.7. LRMS (DART+) *m/z* (%): 397 (100%) $[M+H]^+$.

S5. Synthesis of Glycidyl palmitate (10b): In a 100 mL round-bottom flask, 4.10 g of 98% palmitic acid (**9b**) (15.6 mmol) and 8.18 g of triphenylphosphine (31.2 mmol) were added. Both reagents were dissolved in 22 mL of THF (previously distilled and dried). Subsequently, 1.20 mL of 96% glycidol (**11**) was added to the reaction flask. The flask was stopped with a rubber stopper, and the system was purged with an inert atmosphere (argon) for 1 minute. Finally, DIAD was added dropwise with a syringe for approximately 20 minutes. The reaction was maintained for 18 hours. Once complete, the solvent was evaporated from the reaction mixture under reduced pressure. The product obtained was a yellowish amorphous solid, which was added to a flask containing a 95:5 hexane:ethyl acetate mixture to precipitate as much triphenylphosphine oxide as possible. The reaction mixture was

decanted, and the liquid phase was impregnated with silica gel. The solvent was then removed by distillation under reduced pressure. Column chromatography was performed using a 95:5 hexane:ethyl acetate system. After column chromatography, 4.23 g of a whitish solid were obtained, representing an 86% yield.

Spectroscopic summary: ^{1}H NMR ($CDCl_3$, 300 MHz, 25 °C) δ = 0.87 (t, *J* = 6 Hz, 3H, C*H₃*), 1.24 (s, 24H, C*H₂*), 1.62 (q, *J* = 6 Hz, 2H, C*H₂*), 2.34 (t, *J* = 6 Hz, 2H, COC*H₂*), 3.58 (dd, *J* = 7.5, 6 Hz, 1H, OC*H*), 3.68 (dd, *J* = 6, 3 Hz, 1H, OC*H*), 3.88 – 3.94 (m, 1H, C*H*), 4.04 – 4.22 (m, 2H). ^{13}C NMR ($CDCl_3$, 75 MHz) δ = 14.1, 22.7, 24.9, 29.1, 29.2, 29.3, 29.4, 29.6, 29.7, 31.9, 34.1, 63.3, 65.1, 70.2, 174.3; IR (ATR): 435, 719, 856, 1170, 1702, 1739, 2848, 2916, 2953 cm^{-1}; LRMS (DART+) *m/z* (%): 313 (100%) $[M+H]^+$.

S6. Synthesis of Glycidyl oleate (10c): A procedure similar to that used for product **10b** was followed. In a 50 mL round-bottom flask, 1.00 g of 95% oleic acid (**9c**) (2.98 mmol) and 1.50 g of triphenylphosphine (5.66 mmol) were added. Both reagents had been dissolved in 6 mL of THF (previously distilled and dried). Subsequently, 0.38 mL of 96% glycidol (**11**) (5.66 mmol) was added to the reaction flask. The reaction flask was covered with aluminum foil to protect it from light. The reaction was carried out for 16 hours. Once complete, the reaction mixture was evaporated under reduced pressure. The resulting product was a yellowish liquid, which was added to a flask containing a 97:3 hexane:ethyl acetate mixture to precipitate as much triphenylphosphine oxide as possible. The reaction mixture was decanted, and the liquid phase was impregnated onto silica gel. The solvent was removed by distillation under reduced pressure. Column chromatography was performed using a 95:5 hexane:ethyl acetate system. After column chromatography, 1.00 g of a colorless liquid was obtained in an 83% yield.

Spectroscopic summary: ^{1}H NMR ($CDCl_3$, 300 MHz, 25 °C) δ = 0.82 – 0.92 (m, 3H, C*H₃*), 1.15 – 1.40 (m, 40H, 20C*H₂*), 1.62 (p, *J* = 7.3 Hz, 2H, C*H₂*), 2.35 (t, *J* = 7.5 Hz, 2H, C*H₂*), 2.65 (dd, *J* = 4.9, 2.6 Hz, 1H, $C\mathbf{H_a}H_bOCH$), 2.82 – 2.87 (m, 1H, $CH_a\mathbf{H_b}OCH$), 3.21 (dq, *J* = 6.5, 2.9 Hz, 1H, $CH_aH_bOC\mathbf{H}$), 3.91 (dd, *J* = 12.3, 6.4 Hz, 1H, $C\mathbf{H_a}H_bOCO$), 4.42 (dd, *J* = 12.3, 3.1 Hz, 1H, $CH_a\mathbf{H_b}OCO$), 5.31 – 5.38 (m, 2H, HC=CH). ^{13}C NMR ($CDCl_3$, 75 MHz, 25°C) δ = 14.2, 22.8, 24.9, 27.2, 27.3, 29.2, 29.2, 29.4, 29.6, 29.7, 29.8, 29.9, 32.0, 34.1, 44.7, 49.5, 64.9, 76.7, 77.2, 77.6, 129.8, 130.1, 173.6; LRMS (DART+) *m/z* (%): 339 (100%) $[M+H]^+$.

S7. Synthesis of 1,3-Dipalmityl-2-(4-acetyl)ferulyl glyceride (16): In a 50 mL round-bottom flask, 0.100 g of 1,3-dipalmityl glycerol (**7b**) (0.175 mmol), 0.061 g of 4-acetylferulic acid (**8a**) (0.262 mmol), and 0.007 g of DMAP (0.057 mmol) were added. The reagents were dissolved in 5 mL of dry dichloromethane (DCM). The flask was placed at 0°C, and 0.054 g of *N,N*'-dicyclohexylcarbodiimide (DCC, 0.262 mmol) was added. Stirring at 0°C continued for 10 min and subsequently at room temperature for 48 h. Once the reaction was complete, the reaction mixture was filtered and purified by column chromatography using a 95:5 hexane:ethyl acetate system. 0.096 g of a whitish amorphous solid was obtained, giving a yield of 70%.

Spectroscopic summary: ^{1}H NMR ($CDCl_3$, 400 MHz, 25 °C) δ = 0.88 (t, *J* = 6.8 Hz, 6H, $2CH_3$), 1.19 – 1.35 (m, 48H, $24CH_2$), 1.61 (p, *J* = 7.1 Hz, 4H, $2CH_2C\mathbf{H_2}CH_2OCO$), 2.29 – 2.33 (m, 7H, $2C\mathbf{H_2}OCO$, $C\mathbf{H_3}OCO$), 3.87 (s, 3H, $OC\mathbf{H_3}$), 4.26 (dd, *J* = 11.9, 5.9 Hz, 2H, $C\mathbf{H_2}CHOR$), 4.34 (dd, *J* = 11.9, 4.4 Hz, 2H, $C\mathbf{H_2}CHOR$), 5.39 – 5.43 (m, 1H, $CH_2C\mathbf{H}OCH_2$), 6.38 (d, *J* = 15.9 Hz, 1H, $RC{=}C\mathbf{H}CO_2$), 7.06 (d, *J* = 8.0 Hz, 1H, **5'-H**), 7.11 (d, *J* = 1.8 Hz, 1H, **2'-H**), 7.13 (dd, *J* = 8.0, 1.9 Hz, 1H, **6'-H**),

7.66 (d, J = 15.9 Hz, 1H, PhC**H**=CR). ^{13}C NMR ($CDCl_3$, 100 MHz, 25 °C) δ = 14.2, 20.7, 22.8, 25.0, 29.2, 29.4, 29.5, 29.6, 32.0, 34.1, 56.0, 62.2, 68.9, 111.3, 117.5, 121.5, 123.4, 133.2

S8. Synthesis of 1,3-Dipalmityl-2-(3,4-diacetyl)caffeoyl glyceride (18): In a 50 mL round-bottom flask, 0.150 g of 1,3-dipalmityl glycerol (**7b**) (0.263 mmol), 0.104 g of 3,4-diacetylcaffeic acid (**8b**) (0.395 mmol), and 0.012 g of DMAP (0.105 mmol) were added. The reagents were dissolved in 10 mL of dry dichloromethane (DCM). The flask was placed at 0°C, and 0.081 g of *N,N'*-dicyclohexylcarbodiimide (DCC, 0.395 mmol) was added. Stirring at 0°C continued for 10 min, followed by incubation at room temperature for 48 h. Once the reaction was complete, the reaction mixture was filtered and purified by column chromatography using a 9:1:1 hexane:ethyl acetate:DCM system. 0.066 g of a whitish amorphous solid was obtained, giving a yield of 31%.
Spectroscopic summary: ^{1}H NMR ($CDCl_3$, 400 MHz, 25 °C) δ = 0.88 (t, J = 6.7 Hz, 6H, $2CH_3$), 1.18 – 1.35 (m, 48H. $24CH_2$), 1.60 (p, J = 7.2 Hz, 4H, $2CH_2$**CH_2**CH_2OCO), 2.28 – 2.36 (m, 10H, 2C**H_2**OCO, 2C**H_3**OCO), 4.25 (dd, J = 12.0, 6.0 Hz, 2H, C**H_2**CHOR), 4.33 (dd, J = 12.0, 4.3 Hz, 2H, C**H_2**CHOR), 5.39 (tt, J = 5.9, 4.3 Hz, 1H, CH_2C**H**OCH_2), 6.38 (d, J = 16.0 Hz, 1H, RC=C**H**CO_2), 7.23 (d, J = 8.4 Hz, 1H, **5'H**), 7.37 (d, J = 2.0 Hz, 1H, **2′H**), 7.41 (dd, J = 8.4, 2.1 Hz, 1H, **6'H**), 7.64 (d, J = 16.0 Hz, 1H, PhC**H**=CR). ^{13}C NMR ($CDCl_3$,100 MHz, 25°C) δ = 14.3, 20.8, 22.8, 25.0, 29.3, 29.4, 29.5, 29.6, 29.7, 29.8, 29.8, 32.1, 34.2, 62.2, 69.6, 118.6, 123.0, 124.1, 126.7, 142.6, 143.8, 144.0, 165.7, 168.2, 173.5.

S9. Synthesis of 1,3-Dipalmityl-2-cinnamyl glyceride (20): In a 50 mL round-bottom flask, 0.100 g of 1,3-dipalmityl glycerol (**7b**) (0.175 mmol), 0.038 g of cinnamic acid (**8c**) (0.262 mmol), and 0.009 g of DMAP (0.070 mmol) were added. The reagents were dissolved in 7 mL of dry dichloromethane (DCM). The flask was placed at 0°C, and 0.054 g of *N,N'*-dicyclohexylcarbodiimide (DCC, 0.262 mmol) was added. Stirring at 0°C continued for 10 min, followed by stirring at room temperature for 48 h. After the reaction was complete, the reaction mixture was filtered and purified by column chromatography using a 98:2 hexane:ethyl acetate system. 0.084 g of a whitish amorphous solid was obtained, giving a yield of 69%.
Spectroscopic summary: ^{1}H NMR ($CDCl_3$, 400 MHz, 25 °C) δ = 0.87 (t, J = 6.7 Hz, 6H, $2CH_3$), 1.19 – 1.34 (m, 48H, 24 CH_2), 1.61 (p, J = 7.4 Hz, 4H, $2CH_2$**CH_2**CH_2OCO), 2.32 (t, J = 7.5 Hz, 4H, 2C**H_2**OCO), 4.26 (dd, J = 11.9, 6.0 Hz, 2H, C**H_2**CHOR), 4.34 (dd, J = 11.9, 4.3 Hz, 2H, C**H_2**CHOR), 5.40 (tt, J = 6.0, 4.3 Hz, 1H, CH_2C**H**OCH_2), 6.43 (d, J = 16.0 Hz, 1H, RC=C**H**CO_2), 7.35 – 7.41 (m, 3H, **3'H, 4'H, 5'H**), 7.49 – 7.56 (m, 2H, **2'H, 6'H),** 7.71 (d, J = 16.0 Hz, 1H, PhC**H**=CR). ^{13}C NMR ($CDCl_3$,100 MHz, 25°C) δ = 14.2, 22.8, 25.0, 29.2, 29.4, 29.5, 29.6, 29.7, 29.7, 29.8, 29.8, 29.8, 32.0, 34.2, 62.2, 69.4, 117.4, 128.3, 129.0, 130.6, 134.3, 146.0, 166.0, 173.4.

S10. Synthesis of 1,3-Dioleoyl-2-(4-acetyl)ferulyl glyceride (21): In a 50 mL round-bottom flask, 0.100 g of 1,3-dioleyl glycerol (**7c**) (0.161 mmol), 0.057 g of 4-acetylferulic acid (**8a**) (0.241 mmol), and 0.008 g of DMAP (0.064 mmol) were added. The reagents were dissolved in 15 mL of dry dichloromethane (DCM). The flask was placed at 0°C, and 0.049 g of *N,N'*-dicyclohexylcarbodiimide (DCC, 0.241 mmol) was added. Stirring at 0°C continued for 10 min and subsequently at room temperature for 48 h. Once the reaction was complete, the reaction mixture was filtered and purified

by column chromatography using a 95:5 hexane:ethyl acetate system. 0.079 g of a yellowish translucent liquid was obtained, giving a yield of 61%.

Spectroscopic summary: ^{1}H NMR ($CDCl_3$, 400 MHz, 25 °C) δ = 0.87 (t, *J* = 6.5 Hz, 2H, $2CH_3$), 1.19 – 1.39 (m, 40H, $20CH_2$), 1.54 – 1.66 (m, 4H, $2CH_2C\mathbf{H_2}CH_2OCO$), 1.99 (q, *J* = 6.4 Hz, 8H, $2C\mathbf{H_2}CH{=}CHC\mathbf{H_2}$), 2.27 – 2.38 (m, 7H, 2 $C\mathbf{H_2}OCO$, $C\mathbf{H_3}OCO$), 4.26 (dd, *J* = 11.9, 6.0 Hz, 2H, $C\mathbf{H_2}CHOR$), 4.33 (dd, *J* = 12.0, 4.3 Hz, 2H, $C\mathbf{H_2}CHOR$), 5.33 (m, 4H, C**H**=C**H**), 5.40 (tt, *J* = 6.1, 4.3 Hz, 1H, $CH_2C\mathbf{H}OCH_2$), 6.38 (d, *J* = 15.9 Hz, 1H, $RC{=}C\mathbf{H}CO_2$), 7.05 (d, *J* = 8.0 Hz, 1H, **5'H**), 7.11 (d, *J* = 2.2 Hz, 1H, **2'H**), 7.13 (dd, *J* = 8.0, 2.1 Hz, 1H, **6'H**), 7.66 (d, *J* = 16.0 Hz, 1H, PhC**H**=CHR). ^{13}C NMR ($CDCl_3$, 100 MHz, 25 °C) δ = 14.2, 20.8, 22.8, 25.0, 27.3, 27.3, 29.2, 29.3, 29.4, 29.6, 29.8, 29.9, 32.0, 34.2, 56.0, 62.3, 69.5, 111.4, 117.6, 121.5, 123.4, 129.8, 130.1, 133.2, 141.9, 145.3, 151.6, 165.9, 168.8, 173.4.

S11. Synthesis of 1,3-Dioleoyl-2-(3,4-diacetyl)caffeoyl glyceride (22): In a 50 mL dry round-bottom flask, 0.100 g of 1,3-dioleyl glycerol (**7c**) (0.161 mmol), 0.063 g of 3,4-diacetylcaffeic acid (**8b**) (0.241 mmol), and 0.006 g of DMAP (0.048 mmol) were added. The reagents were dissolved in 5 mL of dry dichloromethane (DCM). The flask was placed at 0°C, and 0.049 g of *N,N'*-dicyclohexylcarbodiimide (DCC, 0.241 mmol) was added. Stirring at 0°C continued for 10 min, followed by incubation at room temperature for 48 h. After the reaction was complete, the reaction mixture was filtered and purified by column chromatography using a 95:5 hexane:ethyl acetate system. 0.047 g of a yellowish translucent liquid was obtained, giving a yield of 34%.

Spectroscopic summary: ^{1}H NMR ($CDCl_3$, 400 MHz, 25 °C) δ = 0.82 – 0.92 (m, 6H, $2CH_3$), 1.22 – 1.36 (m, 40H, $20CH_2$), 1.55 – 1.65 (m, 4H, $2CH_2C\mathbf{H_2}CH_2OCO$), 2.00 (q, *J* = 6.5 Hz, 8H, $2C\mathbf{H_2}CH{=}CHC\mathbf{H_2}$), 2.25 – 2.39 (m, 10H, $2C\mathbf{H_2}OCO$, $2C\mathbf{H_3}OCO$), 4.24 (dd, *J* = 12.0, 6.0 Hz, 2H, $C\mathbf{H_2}CHOR$), 4.33 (dd, *J* = 11.9, 4.3 Hz, 2H, $C\mathbf{H_2}CHOR$), 5.31 – 5.35 (m, 4H, C**H**=C**H**), 5.38 (tt, *J* = 6.0, 4.3, 1H, $CH_2C\mathbf{H}OCH_2$), 6.38 (d, *J* = 16.0 Hz, 1H,$PhCH{=}C\mathbf{H}CO_2$), 7.23 (d, *J* = 8.4 Hz, 1H, **5'H**), 7.37 (d, *J* = 2.0 Hz, 1H, **2'H**), 7.40 (dd, *J* = 8.6, 2.1 Hz, 1H, **6'H**), 7.64 (d, *J* = 16.0 Hz, 1H, $PhC\mathbf{H}{=}CHCO_2$). ^{13}C NMR ($CDCl_3$, 100 MHz, 25 °C) δ = 14.2, 20.7, 20.8, 22.8, 25.0, 27.3, 27.3, 29.2, 29.2, 29.3, 29.5, 29.7, 29.8, 29.9, 32.0, 34.2, 62.3, 69.6, 118.6, 123.0, 124.1, 126.6, 129.9, 130.1, 133.15, 142.6, 143.9, 144.0, 165.7, 168.1, 168.1, 173.5.

S12. Synthesis of 1,3-Dioleoyl-2-cinnamyl glyceride (23): In a 50 mL round-bottom flask, 0.100 g of 1,3-dioleyl glycerol (**7c**) (0.161 mmol), 0.036 g of cinnamic acid (**8c**) (0.241 mmol), and 0.008 g of DMAP (0.064 mmol) were added. The reagents were dissolved in 5 mL of dry dichloromethane (DCM). The flask was placed at 0°C, and 0.050 g of *N,N'*-dicyclohexylcarbodiimide (DCC, 0.241 mmol) was added. Stirring at 0°C continued for 10 min and then at room temperature for 48 h. Once the reaction was complete, the reaction mixture was filtered and purified by column chromatography using a 97:3 hexane:ethyl acetate system. 0.096 g of a whitish amorphous solid was obtained, giving a yield of 79%.

Spectroscopic summary: ^{1}H NMR ($CDCl_3$, 400 MHz, 25 °C) δ = 0.88 (t, *J* = 6.6 Hz, 6H, $2CH_3$), 1.19 – 1.38 (m, 40H, $20CH_2$), 1.57 – 1.65 (m, 4H, $2CH_2C\mathbf{H_2}CH_2OCO$), 1.93 – 2.10 (m, 8H, $2C\mathbf{H_2}CH{=}CHC\mathbf{H_2}$), 2.33 (t, *J* = 7.5 Hz, 4H, $2C\mathbf{H_2}OCO$), 4.26 (dd, *J* = 11.9, 6.0 Hz, 2H,), 4.34 (dd, *J* = 11.9, 4.3 Hz, 2H, $C\mathbf{H_2}CHOR$), 5.29 – 5.36 (m, 4H, C**H**=C**H**), 5.41 (tt, *J* = 6.0, 4.3 Hz, 1H, $CH_2C\mathbf{H}OCH_2$), 6.44 (d, *J* = 16.0 Hz, 1H, $PhCH{=}C\mathbf{H}CO_2$), 7.34 – 7.44 (m, 3H, **3'H, 4'H, 5'H**), 7.51

– 7.55 (m, 2H, **2'H,6'H**), 7.71 (d, *J* = 16.0 Hz, 1H, PhC**H**=CHCO$_2$). ^{13}C NMR (CDCl$_3$, 100 MHz, 25 °C) δ = 14.2, 22.8, 25.0, 27.3, 27.4, 29.2, 29.2, 29.3, 29.5, 29.7, 29.8, 29.9, 32.0, 34.2, 62.3, 69.4, 76.8, 77.2, 77.5, 117.2, 117.4, 128.3, 129.1, 129.9, 130.1, 130.7, 134.3, 146.0, 166.1, 173.5.

S13. Synthesis of 1,3-Dibehenyl-2-(4-acetyl)ferulyl glyceride (12): In a 50 mL round-bottom flask, 0.100 g of 1,3-dibehenyl glycerol (**7a**), 0.048 g of 4-acetylferulic acid (**8a**), and 0.005 g of DMAP were added. The reagents were dissolved in 15 mL of dry dichloromethane (DCM). The flask was placed at 0°C, and 0.043 g of *N,N'*-dicyclohexylcarbodiimide (DCC) was added. Stirring at 0°C continued for 10 min and then at room temperature for 48 h. After the reaction was complete, the reaction mixture was filtered and purified by column chromatography using a 9:1 hexane:ethyl acetate system. 0.091 g of a whitish amorphous solid was obtained, giving a 71% yield.
Spectroscopic summary**:** ^{1}H NMR (CDCl$_3$, 400 MHz, 25 °C) δ = 0.87 (t, *J* = 6.7 Hz, 6H, 2CH$_3$), 1.13 – 1.34 (m, 72H, 36CH$_2$), 1.60 (p, *J* = 7.4 Hz, 4H, CH$_2$C**H$_2$**CH$_2$OCO), 2.25 – 2.36 (m, 7H, 2 C**H$_2$**OCO, 1 C**H$_3$**OCO), 4.25 (dd, *J* = 11.9, 5.9 Hz, 2H, C**H$_2$**CHOR), 4.33 (dd, *J* = 11.9, 4.4 Hz, 2H, C**H$_2$**CHOR), 5.35 – 5.43 (m, 1H, CH$_2$C**H**OCH$_2$), 6.37 (d, *J* = 15.9 Hz, 1H, PhC=C**H**CO$_2$), 7.05 (d, *J* = 7.9 Hz, 1H, **5'H**), 7.10 (d, *J* = 2.0 Hz, 1H**, 2'H),** 7.12 (dd, *J* = 7.9, 1.9 Hz, 1H, **6'H**), 7.66 (d, *J* = 16.0 Hz, 1H, PhC**H**=CHCO$_2$). ^{13}C NMR (CDCl$_3$, 100 MHz, 25°C) δ = 14.2, 20.8, 22.8, 25.0, 29.2, 29.4, 29.5, 29.6, 29.7, 29.8, 29.8, 32.1, 34.2, 56.0, 62.2, 69.5, 77.2, 111.4, 117.5, 121.6, 123.4, 133.2, 141.8, 145.3, 151.6, 165.9, 168.8, 173.5.

S14. Synthesis of 1,3-Dibehenyl-2-(3,4-diacetyl)caffeoyl glyceride (14): In a 50 mL round-bottom flask, 0.100 g of 1,3-dibehenyl glycerol (**7a**) (0.135 mmol), 0.053 g of 3,4-diacetylcaffeic acid (**8b**) (0.203 mmol), and 0.007 g of DMAP (0.054 mmol) were added. The reagents were dissolved in 15 mL of dry dichloromethane (DCM). The flask was placed at 0°C, and 0.042 g of *N,N'*-dicyclohexylcarbodiimide (DCC, 0.203 mmol) was added. Stirring at 0°C continued for 10 min and subsequently at room temperature for 48 h. Once the reaction was complete, the reaction mixture was filtered and purified by column chromatography using a 95:5 hexane:ethyl acetate system. 0.024 g of a whitish amorphous solid was obtained, giving a yield of 26%.
Spectroscopic summary: ^{1}H NMR (CDCl$_3$, 400 MHz, 25 °C) δ = 0.88 (t, *J* = 6,6 Hz, 6H, 2CH$_3$), 1.21 – 1.34 (m, 72H, 36CH$_2$), 1.61 (p, *J* = 7.5 Hz, 4H, CH$_2$C**H$_2$**CH$_2$OCO), 2.30 – 2.35 (m, 10H, 2 C**H$_2$**OCO,2 C**H$_3$**OCO), 4.25 (dd, *J* = 12.0, 6.0 Hz, 2H, C**H$_2$**CHOR), 4.33 (dd, *J* = 11.9, 4.3 Hz, 2H, C**H$_2$**CHOR), 5.39 (m, 1H, CH$_2$C**H**OCH$_2$), 6.38 (d, *J* = 15.9 Hz, 1H, RC=C**H**CO$_2$), 7.23 (d, *J* = 8.3 Hz, 1H, **5'-H**), 7.37 (d, *J* = 2.0 Hz, 1H, **2'H**), 7.41 (dd, *J* = 8.5, 2.0 Hz, 1H, **6'H**), 7.64 (d, *J* = 16.0 Hz, 1H, PhC**H**=CR). ^{13}C NMR (CDCl$_3$, 100 MHz, 25°C) δ = 14.2, 20.7, 20.8, 22.8, 25.0, 29.3, 29.4, 29.5, 29.6, 29.8, 29.8, 29.9, 32.1, 34.2, 62.3, 69.6, 118.6, 123.0, 124.1, 126.6, 133.2, 142.6, 143.9, 144.0, 165.7, 168.1, 168.1, 173.5.

S15. Synthesis of 1,3-Dibehenyl-2-cinnamyl glyceride (15): In a 50 mL round-bottom flask, 0.050 g of 1,3-dibehenyl glycerol (**7a**) (0.067 mmol), 0.015 g of cinnamic acid (**8c**) (0.100 mmol), and 0.002 g of DMAP (0.100 mmol) were added. The reagents were dissolved in 5 mL of dry dichloromethane (DCM). The flask was placed at 0°C, and 0.020 g of *N,N'*-dicyclohexylcarbodiimide (DCC, 0.100 mmol) was added. Stirring at 0°C continued for 10 min and then at room temperature for 48 h. Once the reaction was complete, the reaction mixture was filtered and purified by column chromatography

using a 98:2 hexane:ethyl acetate system. 0.040 g of a whitish amorphous solid was obtained, giving a yield of 70%.
Spectroscopic summary: ^{1}H NMR ($CDCl_3$, 400 MHz, 25 °C) δ = 0.88 (t, *J* = 6.7 Hz, 2H, $2CH_3$), 1.18 – 1.36 (m, 72H, $36CH_2$), 1.61 (p, *J* = 8.1 Hz, 4H, $2CH_2C\mathbf{H_2}CH_2OCO$), 2.33 (t, *J* = 7.5 Hz, 4H, 2 $CH_2C\mathbf{H_2}OCO$), 4.26 (dd, *J* = 11.9, 5.9 Hz, 2H, $C\mathbf{H_2}CHOR$), 4.34 (dd, *J* = 11.9, 4.3 Hz, 2H, $C\mathbf{H_2}CHOR$), 5.41 (tt, *J* = 5.9, 4.3 Hz, 1H, $CH_2C\mathbf{H}OCH_2$), 6.44 (d, *J* = 16.0 Hz, 1H, $RC{=}C\mathbf{H}CO_2$), 7.37 – 7.42 (m, 3H,**3'H,4'H,5'H**), 7.50 – 7.56 (m, 2H,**2'H,6'H**), 7.71 (d, *J* = 16.0 Hz, 1H, PhC**H**=CR). ^{13}C NMR ($CDCl_3$,100 MHz, 25°C) δ = 14.3, 22.8, 25.0, 29.4, 29.5, 29.6, 29.8, 29.8, 29.8, 29.9, 32.1, 34.2, 62.3, 69.4, 117.4, 128.4, 129.1, 130.7, 134.3, 146.0, 166.1, 173.5.

S16. Synthesis of 1,3-Dipalmityl-2-caffeoyl glyceride (19): In a 25 mL round-bottom flask, 0.050 g of 1,3-dipalmityl-2-(3,4-diacetyl)caffeoyl glyceride (18) (0.061 mmol) was dissolved in 4 mL of a 1:1 mixture of DCM and methanol. Subsequently, 0.021 g of potassium carbonate (0.152 mmol) was added. The reaction was allowed to proceed for 2 hours. The product was purified by column chromatography using an 85:15 hexane/ethyl acetate system as the eluent. The desired compound was obtained as a whitish amorphous solid (0.037 g, 83% yield).
Spectroscopic summary: ^{1}H NMR ($CDCl_3$, 400 MHz, 25 °C) δ = 0.88 (t, *J* = 6.8 Hz, 6H, $2CH_3$), 1.19 – 1.35 (m, 48H, $24CH_2$), 1.61 (p, *J* = 7.3 Hz, 4H, $2CH_2C\mathbf{H_2}CH_2OCO$), 2.33 (t, *J* = 7.5 Hz, 4H, $2CH_2C\mathbf{H_2}OCO$), 4.27 (dd, *J* = 11.9, 5.9 Hz, 2H, $C\mathbf{H_2}CHOR$), 4.34 (dd, *J* = 11.9, 4.4 Hz, 2H, $C\mathbf{H_2}CHOR$), 5.34 – 5.42 (m, 1H, $CH_2C\mathbf{H}OCH_2$), 6.23 (d, *J* = 15.9 Hz, 1H,$PhCH{=}C\mathbf{H}CO_2$), 6.86 (d, *J* = 8.2 Hz, 1H, **5'H**), 6.98 (dd, *J* = 8.2, 2.0 Hz, 1H, **6'H**), 7.07 (d, *J* = 2.0 Hz, 1H, **2'H**), 7.58 (d, *J* = 15.9 Hz, 1H, $PhC\mathbf{H}{=}CHCO_2$). ^{13}C NMR ($CDCl_3$, 100 MHz, 25 °C) δ = 14.3, 22.8, 25.0, 29.3, 29.4, 29.5, 29.6, 29.8, 29.8, 29.8, 32.1, 34.3, 62.4, 69.3, 114.6, 114.8, 115.7, 122.8, 127.5, 144.1, 146.1, 146.7, 166.6, 173.8.

S17. Synthesis of 1,3-Dibehenyl-2-ferulyl glyceride (1): In a 50 mL round-bottom flask, 0.030 g of 1,3-dibehenyl-2-(4-acetyl)ferulyl glyceride (**12**) (0.032 mmol) was dissolved in 7 mL of a 1:1 mixture of DCM and methanol. Subsequently, 0.014 g of potassium carbonate (0.100 mmol) was added. The reaction was allowed to proceed for 3 hours. The product was purified by column chromatography using an 8:2 hexane/ethyl acetate system as the eluent. The desired compound was obtained as a whitish amorphous solid (0.024 g, 86% yield).
Spectroscopic summary: ^{1}H NMR ($CDCl_3$, 300 MHz, 25 °C) δ = 0.88 (t, *J* =6.7 Hz, 6H, $2CH_3$), 1.20 – 1.32 (m, 72H, $36CH_2$), 1.60 (p, *J* = 6.9 Hz, 5H, $2CH_2C\mathbf{H_2}CH_2OCO$ + H_2O coming from $CDCl_3$), 2.32 (t, *J* = 7.4 Hz, 4H, $2CH_2C\mathbf{H_2}OCO$), 4.26 (dd, *J* = 11.8, 5.9 Hz, 2H, $C\mathbf{H_2}CHOR$), 4.33 (dd, *J* = 11.9, 4.4 Hz, 2H, $C\mathbf{H_2}CHOR$), 5.33 – 5.46 (m, 1H, $CH_2C\mathbf{H}OCH_2$), 6.28 (d, *J* = 15.9 Hz, 1H, $PhCH{=}C\mathbf{H}CO_2$), 6.92 (d, *J* = 8.2 Hz, 1H, **5'H**), 7.03 (d, *J* = 1.9 Hz, 1H, **2'H**), 7.08 (dd, *J* = 8.2, 1.9 Hz, 1H, **6'H**), 7.64 (d, *J* = 15.9 Hz, 1H, $PhC\mathbf{H}{=}CHCO_2$). ^{13}C NMR ($CDCl_3$, 100 MHz, 25 °C) δ = 14.3, 22.8, 25.0, 29.3, 29.4, 29.5, 29.6, 29.8, 29.8, 29.9, 32.1, 34.2, 56.1, 62.4, 69.2, 109.5, 114.7, 114.9, 123.5, 126.9, 146.1, 146.9, 148.4, 166.4, 173.5; LRMS (DART+) *m/z*: 914 [M +OH].

2 Spectra (^{1}H and ^{13}C NMR) of the products 7a-c, 10a-c, 16, 18, 20-23, 12, 14-15, and 19..……S18-S50.

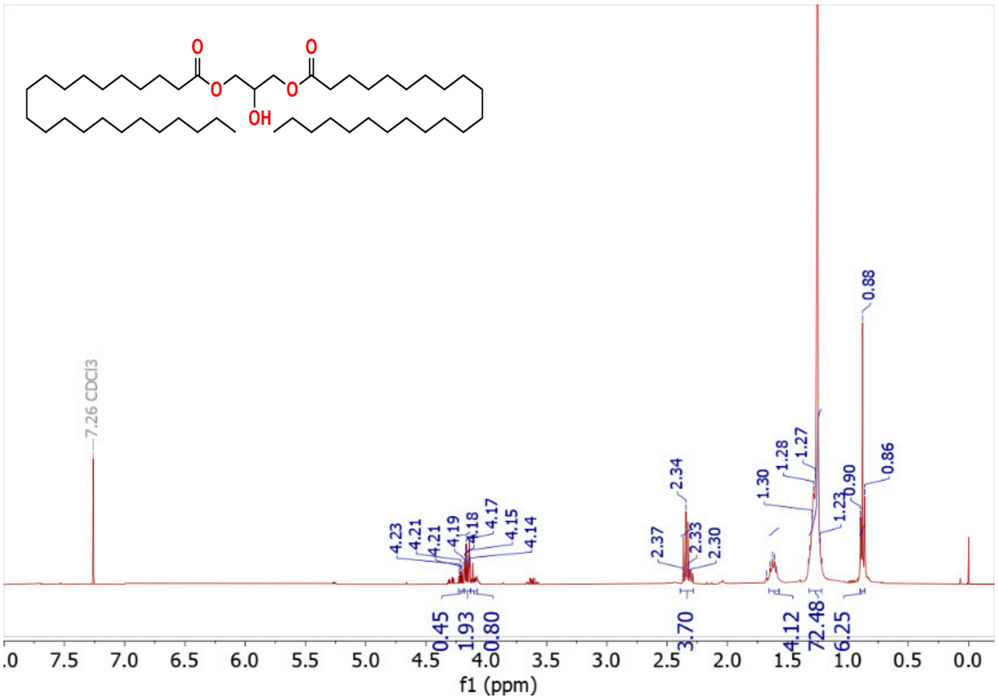

Figure **S18**. ^{1}H NMR Spectra of **7a**.

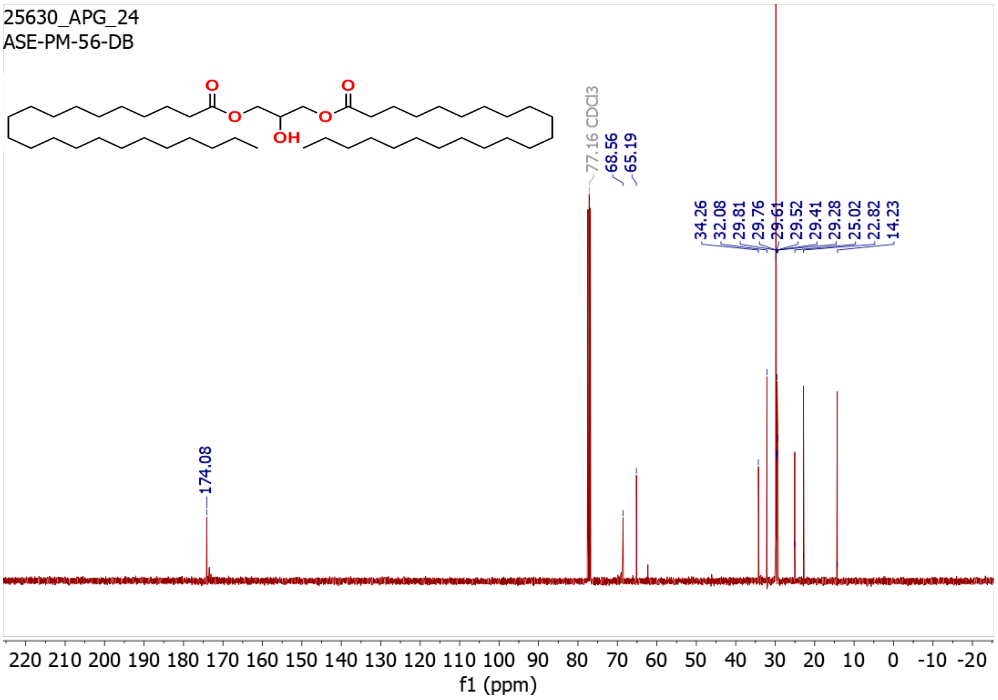

Figure **S19**. ^{13}C NMR Spectra of **7a**.

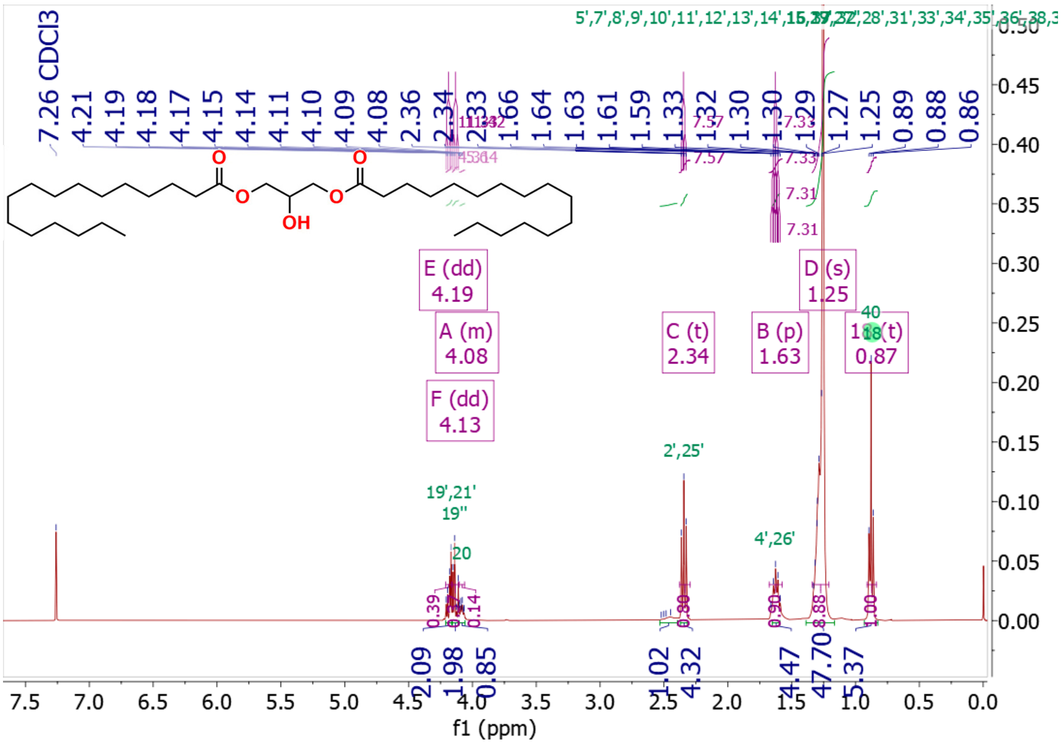

Figure **S20**. ^{1}H NMR Spectra of **7b.**

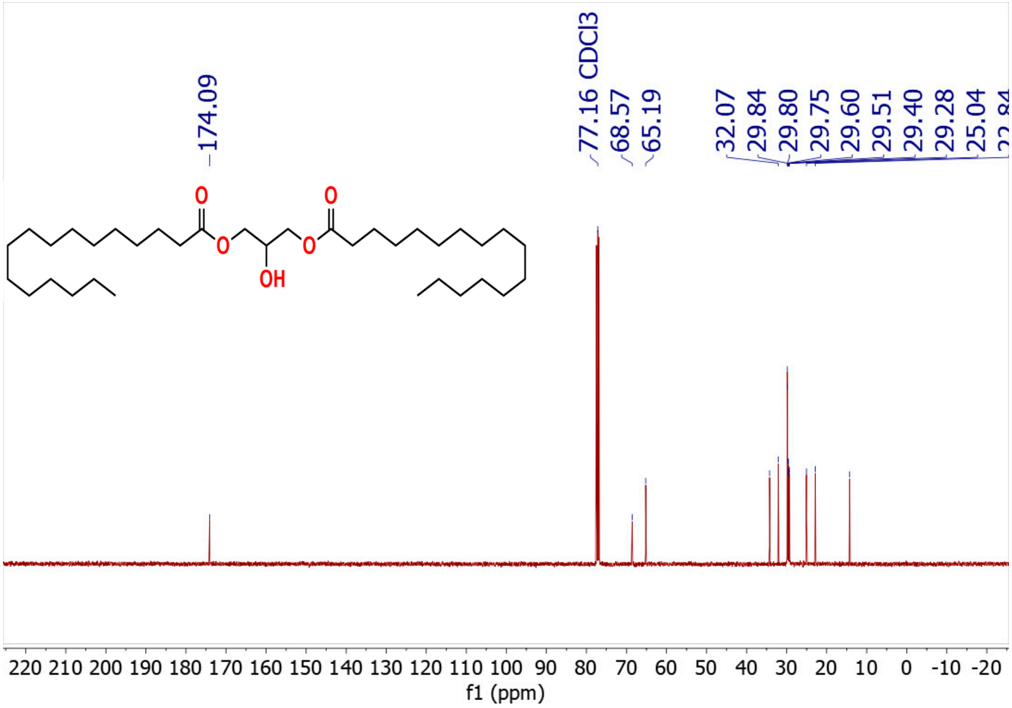

Figure **S21**. ^{13}C NMR Spectra of **7b.**

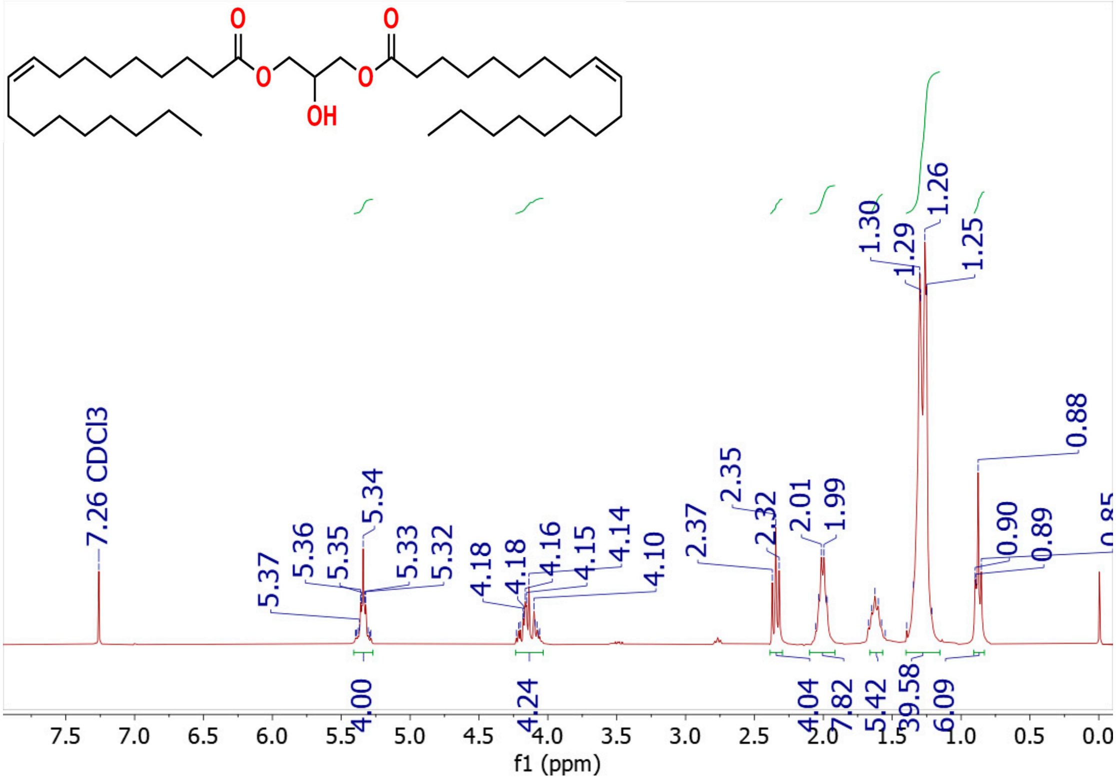

Figure **S22**. ^{1}H NMR Spectra of **7c**.

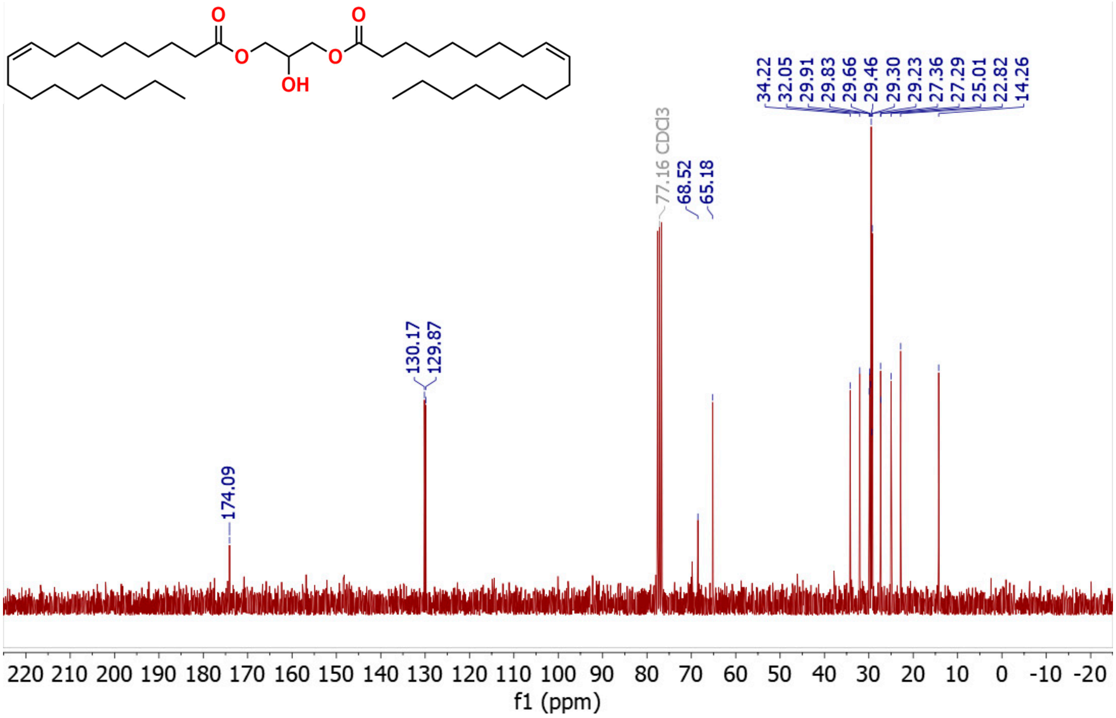

Figure **S23**. ^{13}C NMR Spectra of **7c**.

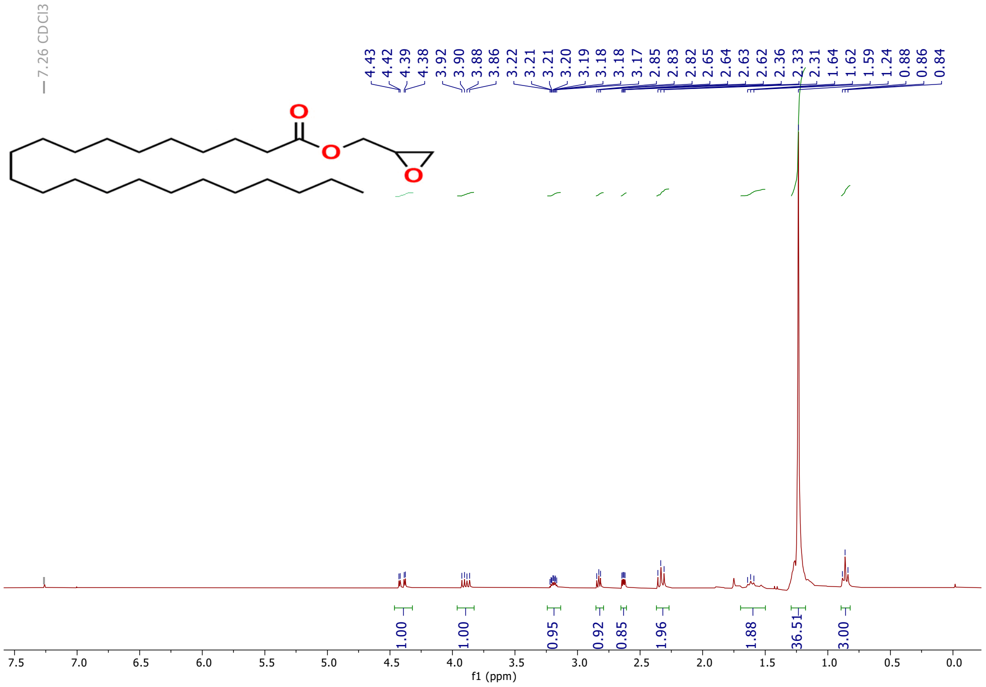

Figure **S24**. ^{1}H NMR Spectra of **10a**.

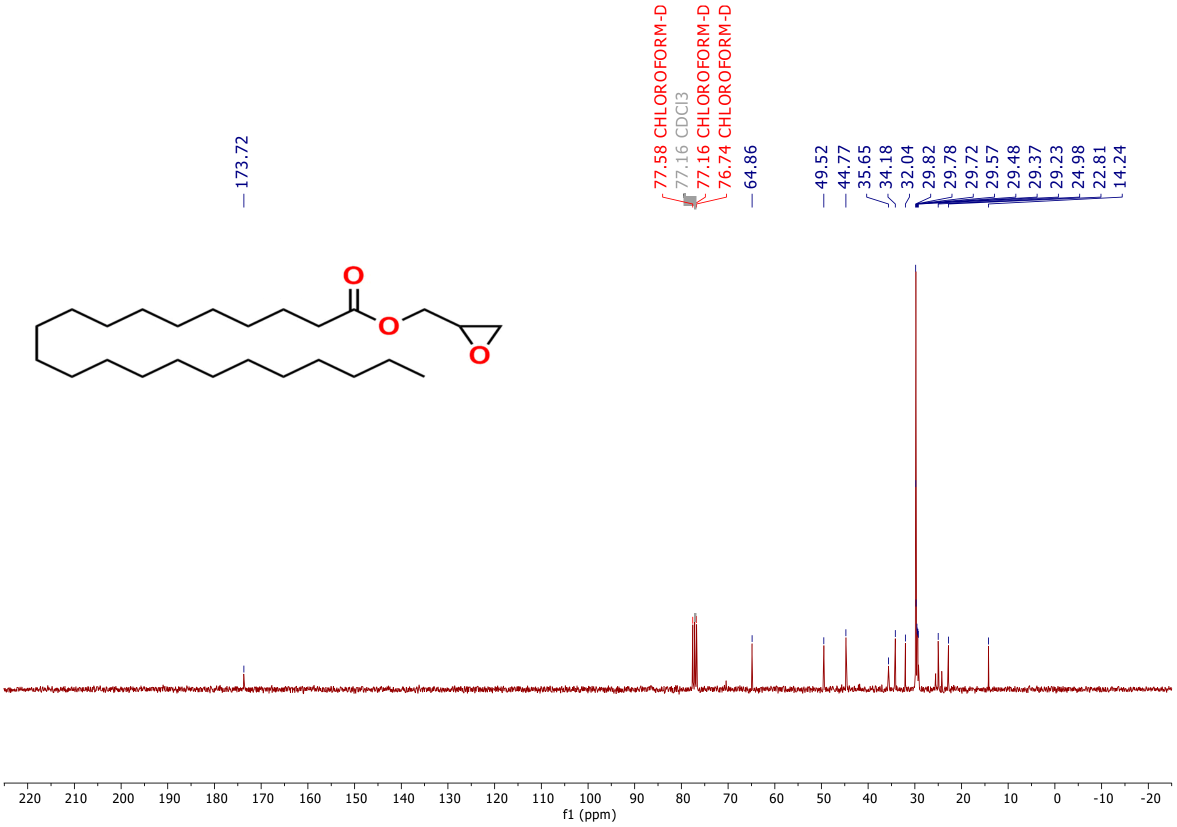

Figure **S25**. ^{13}C NMR Spectra of **10a**.

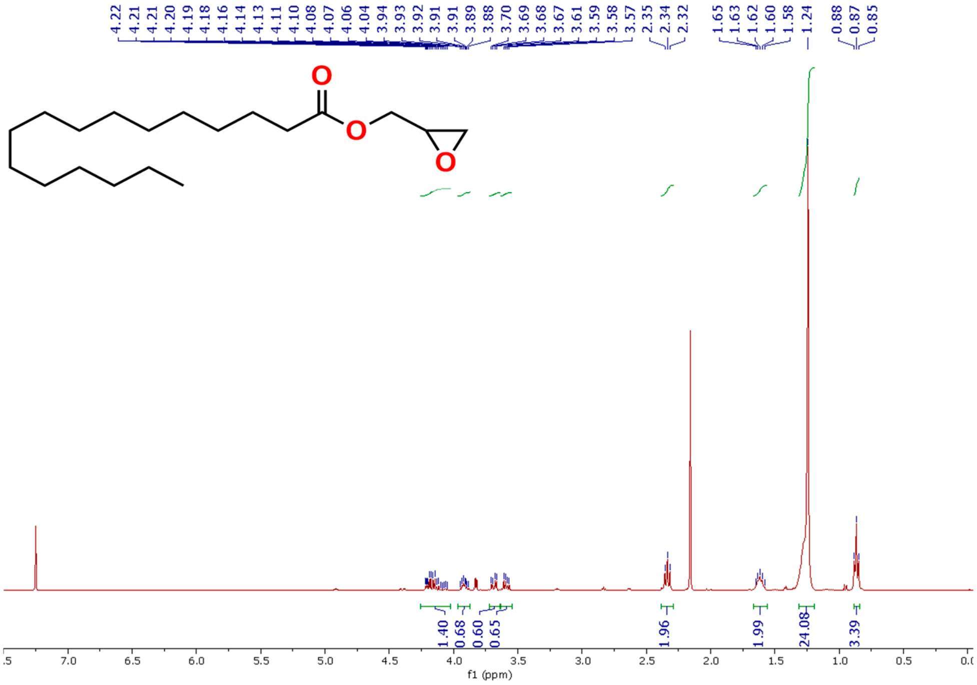

Figure **S26**. ^{1}H NMR Spectra of **10b**.

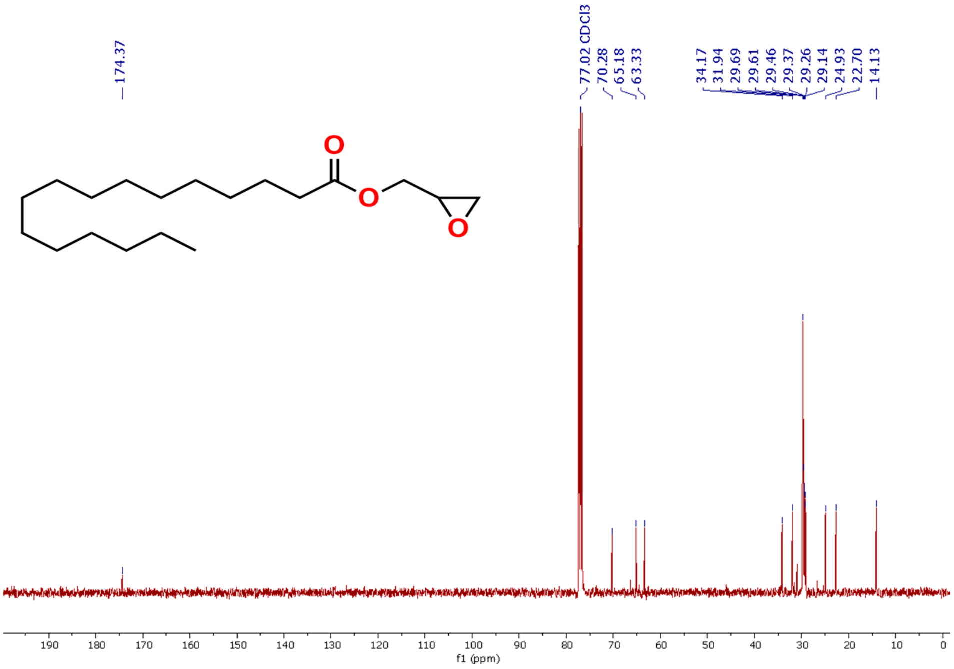

Figure **S27**. ^{13}C NMR Spectra of **10b**.

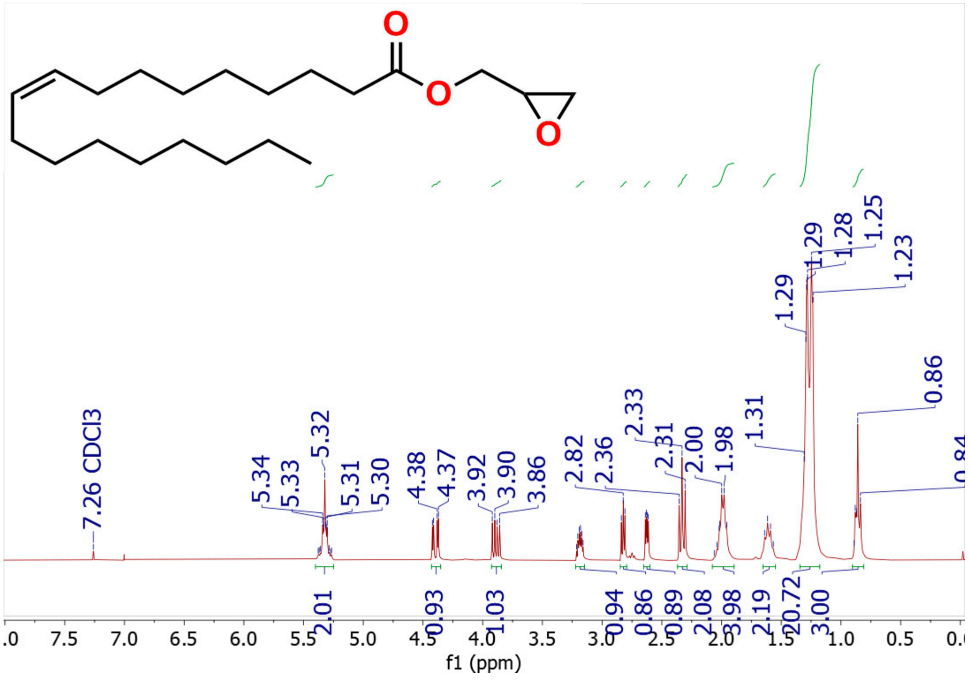

Figure **S28**. ^{1}H NMR Spectra of **10c**.

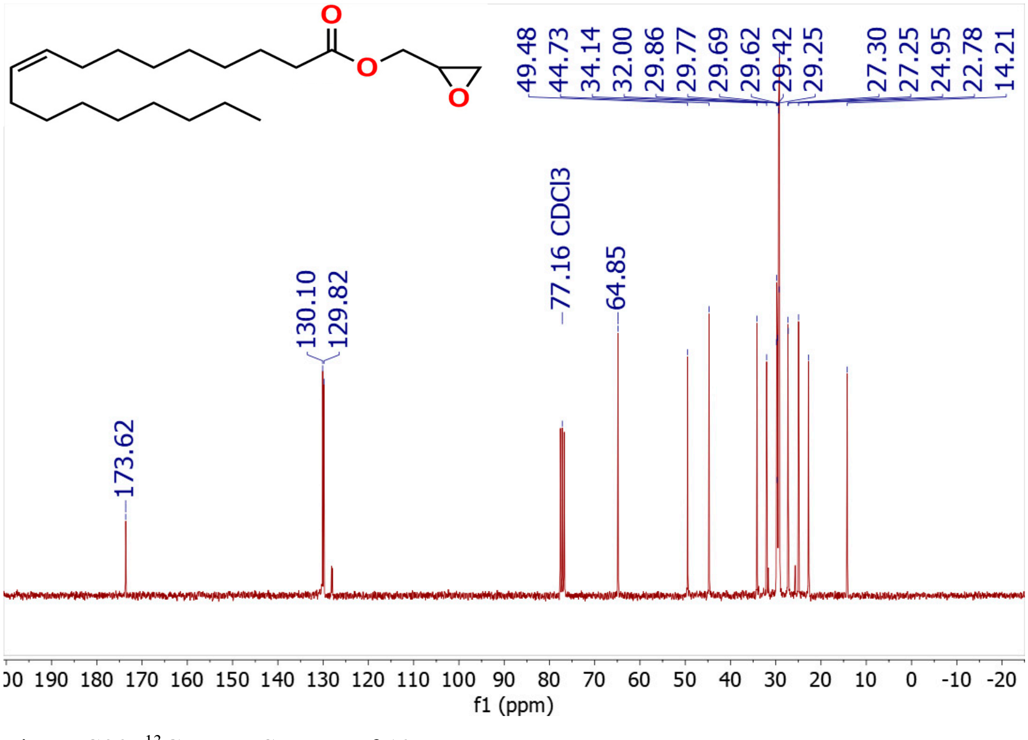

Figure **S29**. ^{13}C NMR Spectra of **10c**.

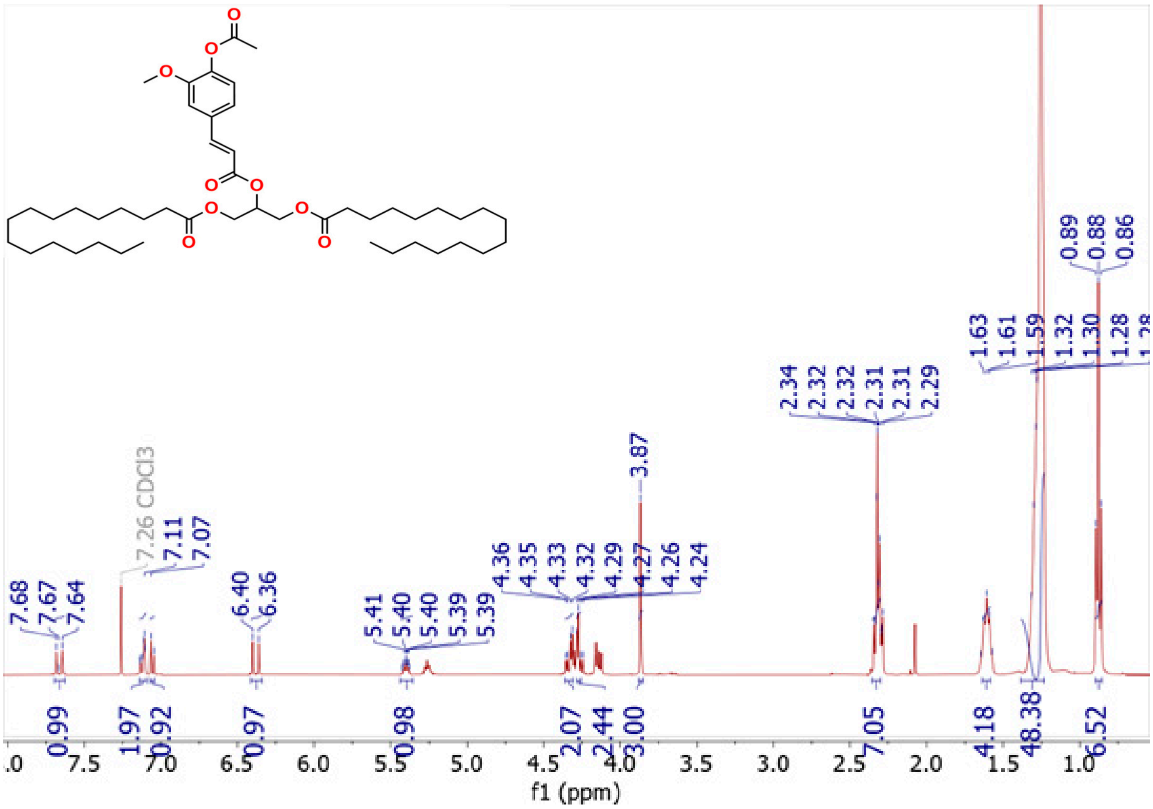

Figure **S30**. ^{1}H NMR Spectra of **16**.

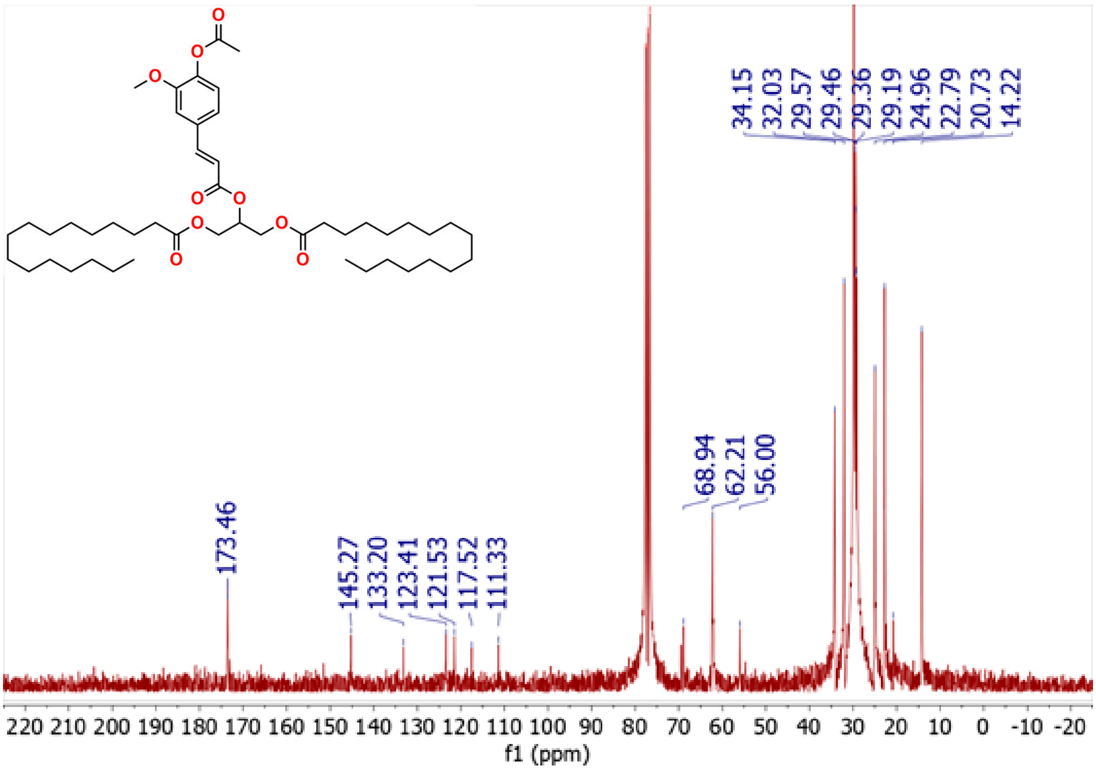

Figure **S31**. ^{13}C NMR Spectra of **16**.

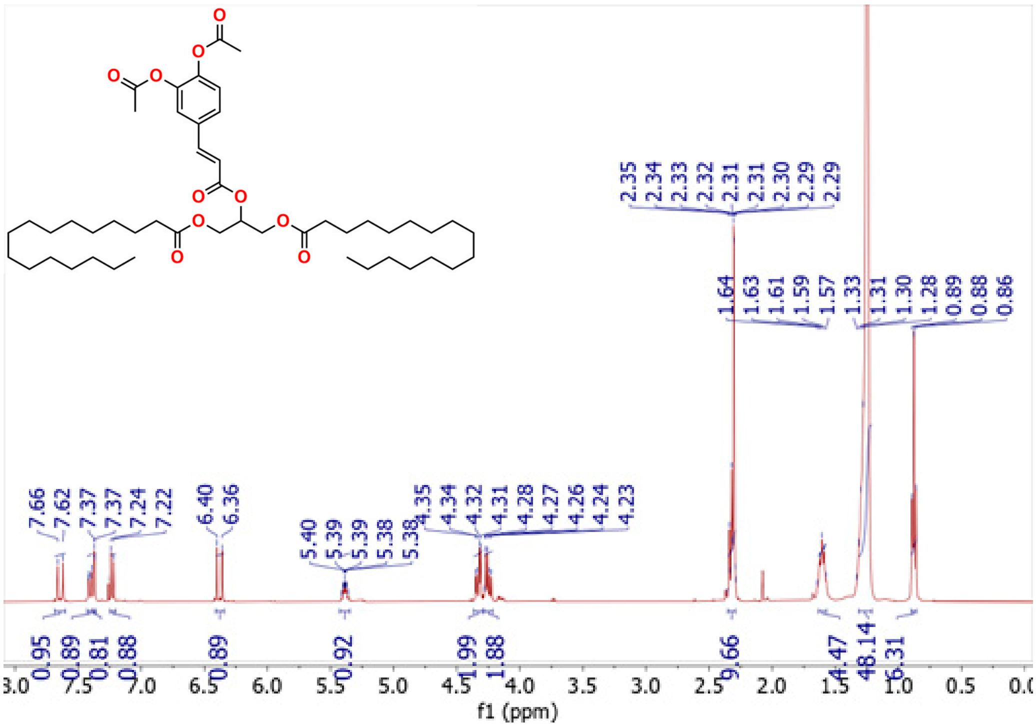

Figure **S32**. ^{1}H NMR Spectra of **18**.

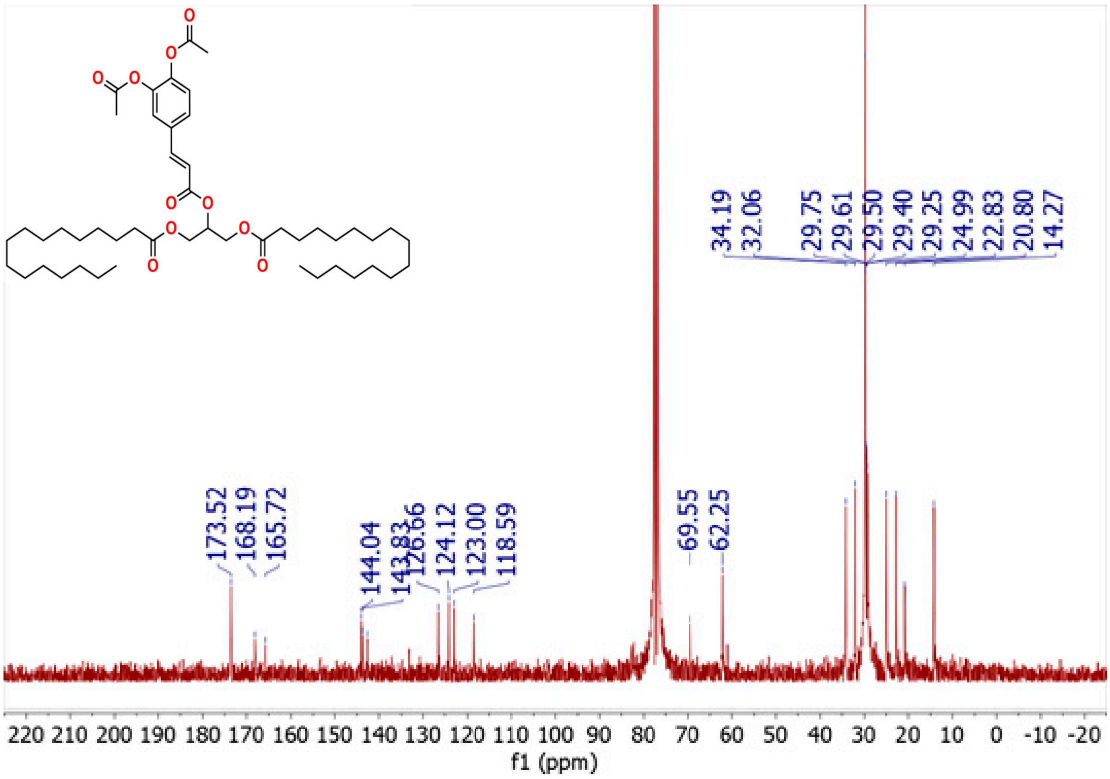

Figure **S33**. ^{13}C NMR Spectra of **18**.

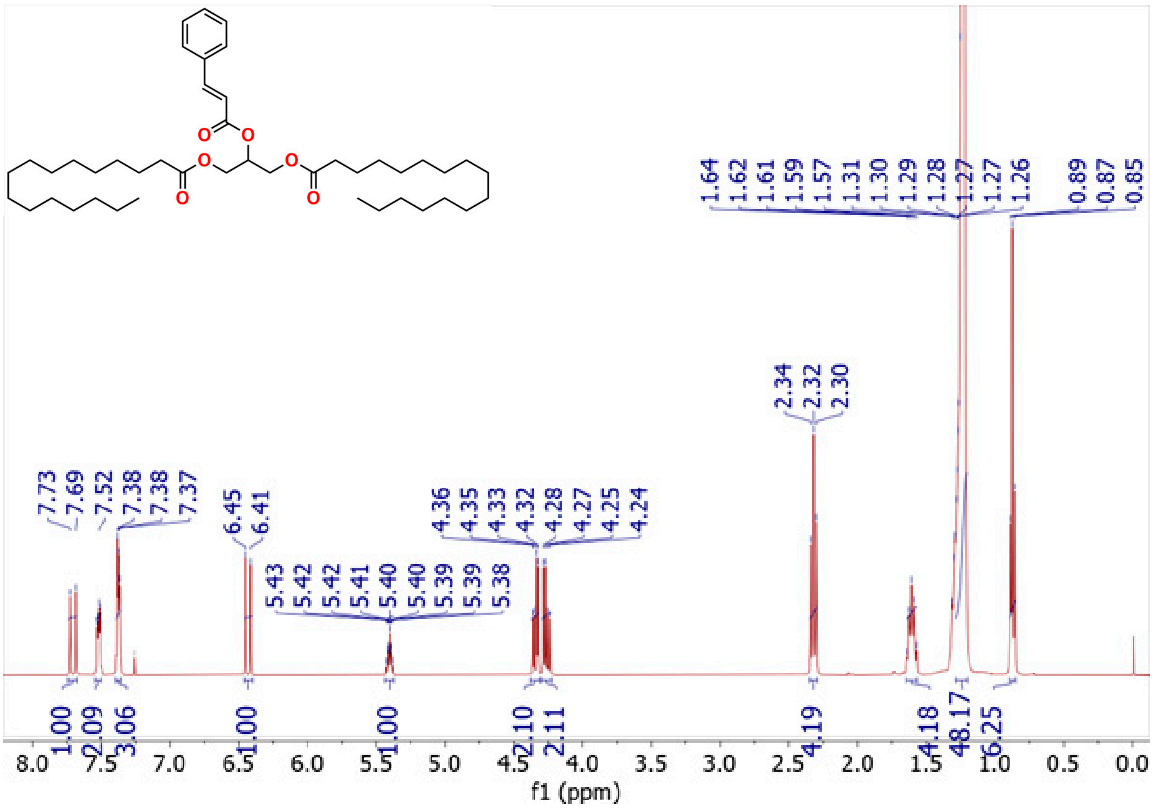

Figure **S34**. ^{1}H NMR Spectra of **20**.

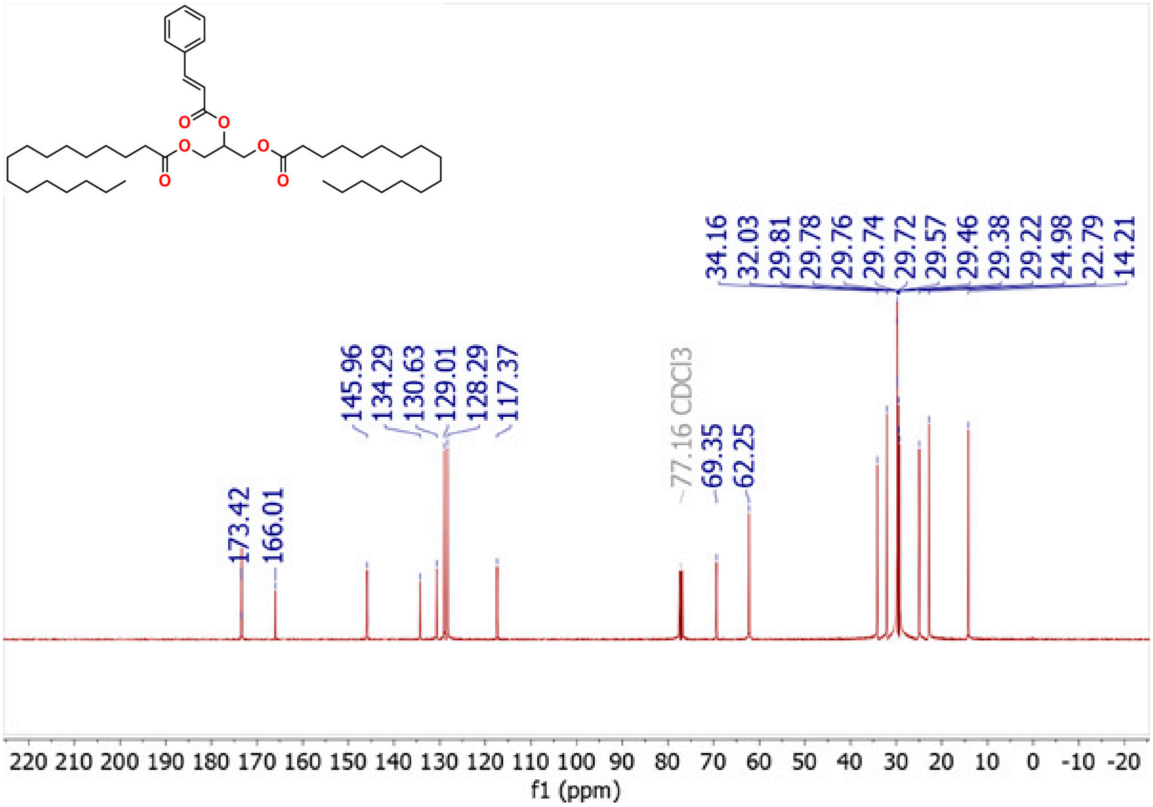

Figure **S35**. ^{13}C NMR Spectra of **20**.

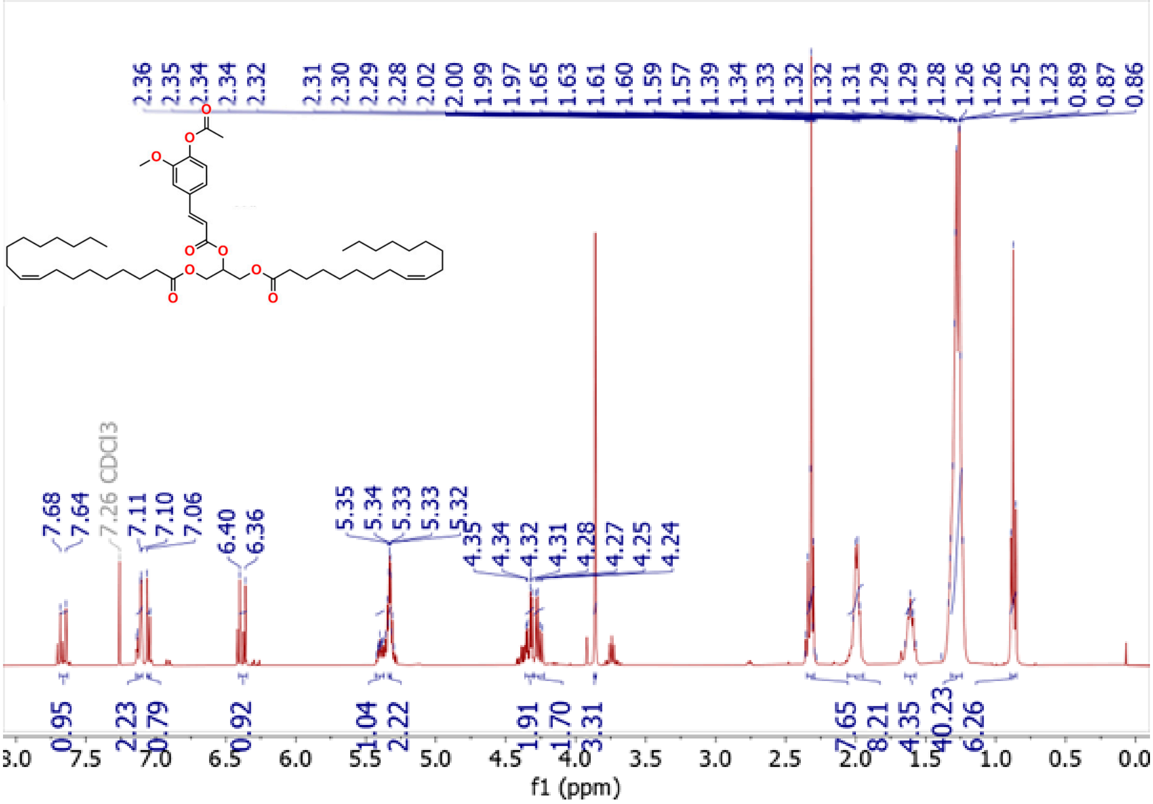

Figure **S36.** ^{1}H NMR Spectra of **21**.

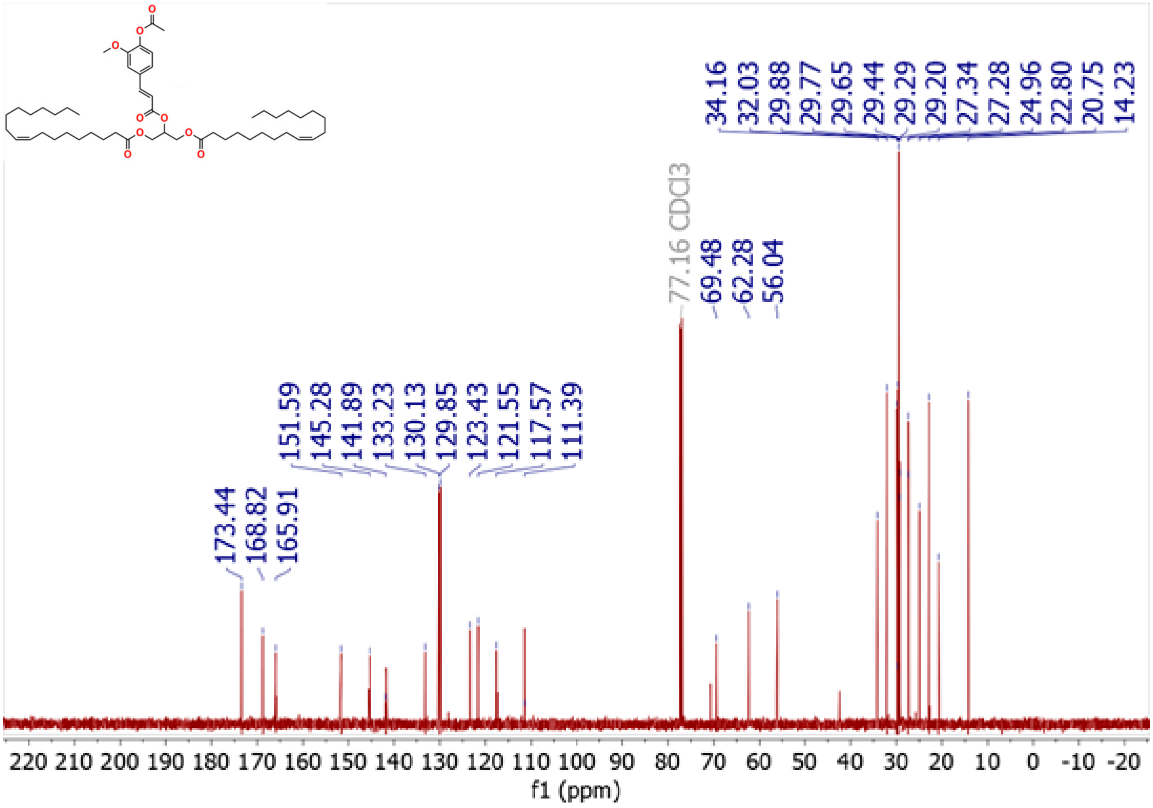

Figure **S37**. ^{13}C NMR Spectra of **21**.

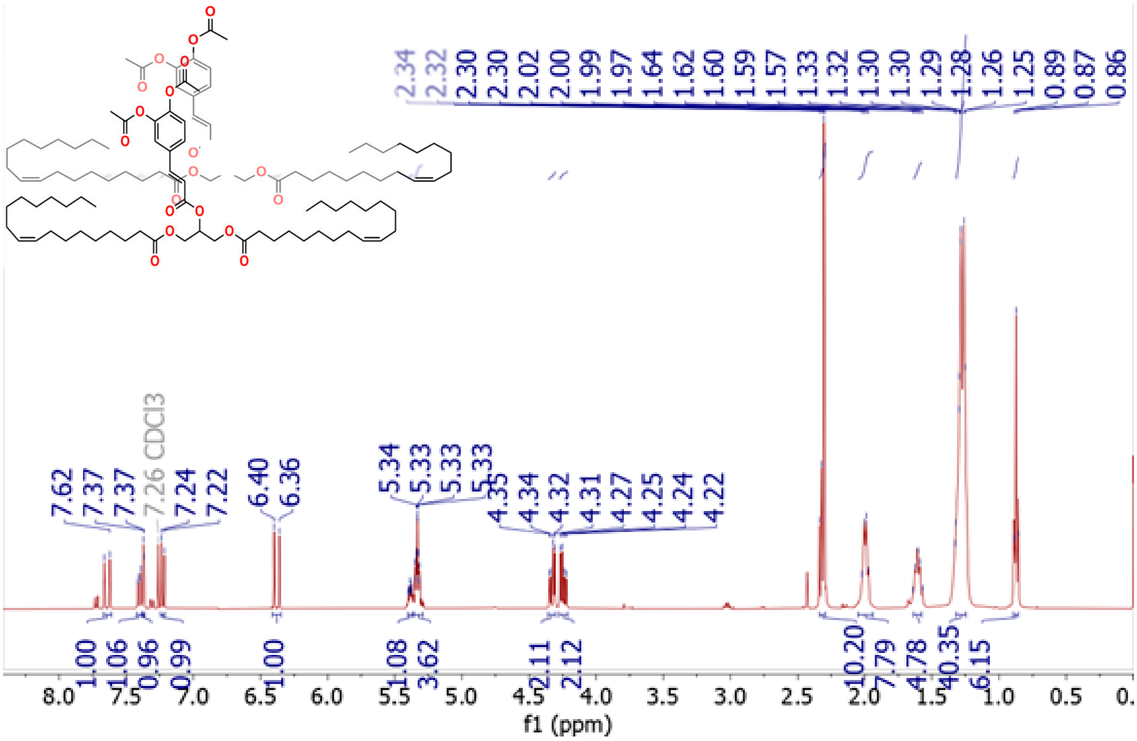

Figure **S38**. ^{1}H NMR Spectra of **22**.

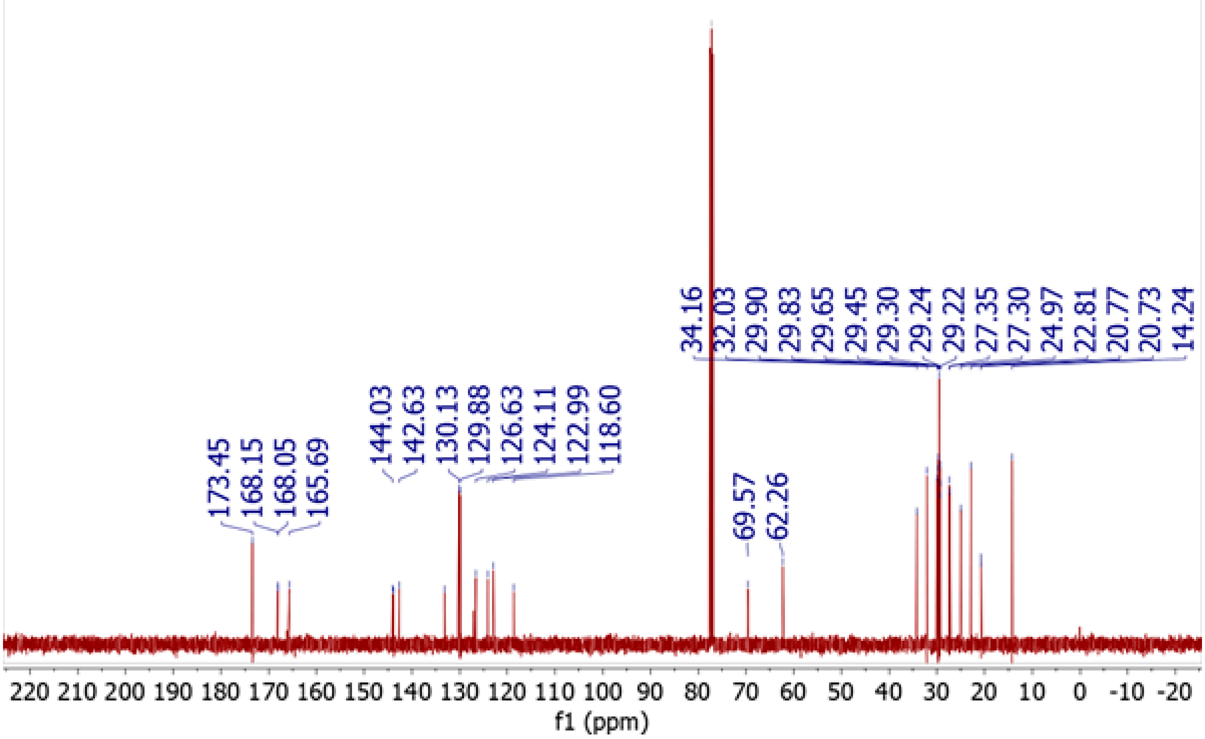

Figure **S39**. ^{13}C NMR Spectra of **22**.

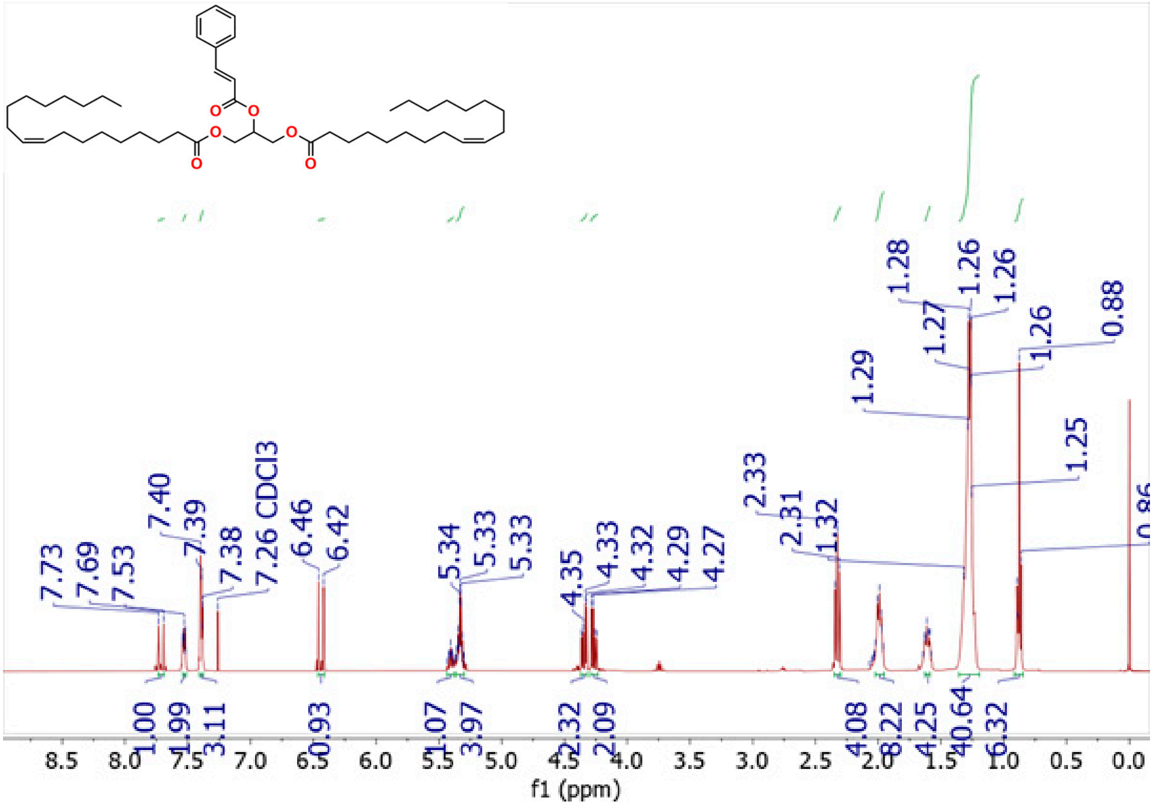

Figure **S40**. ^{1}H NMR Spectra of **23**.

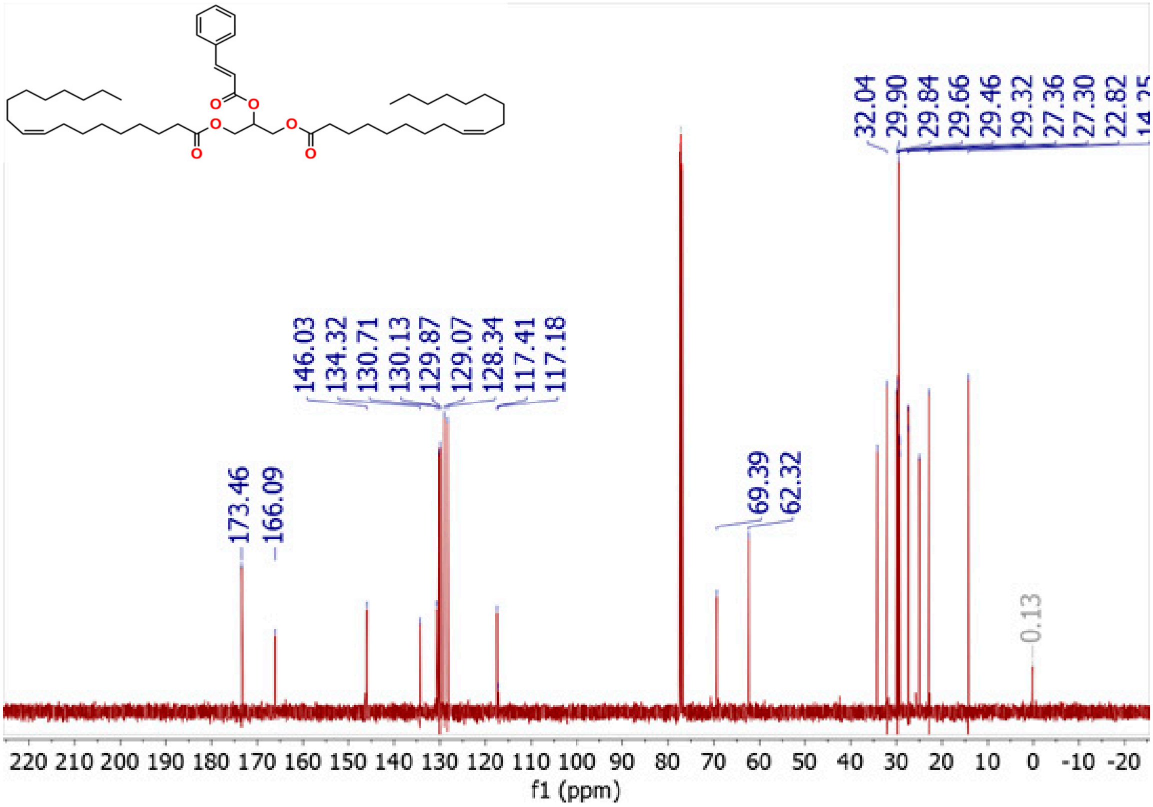

Figure **S41**. ^{13}C NMR Spectra of **23**.

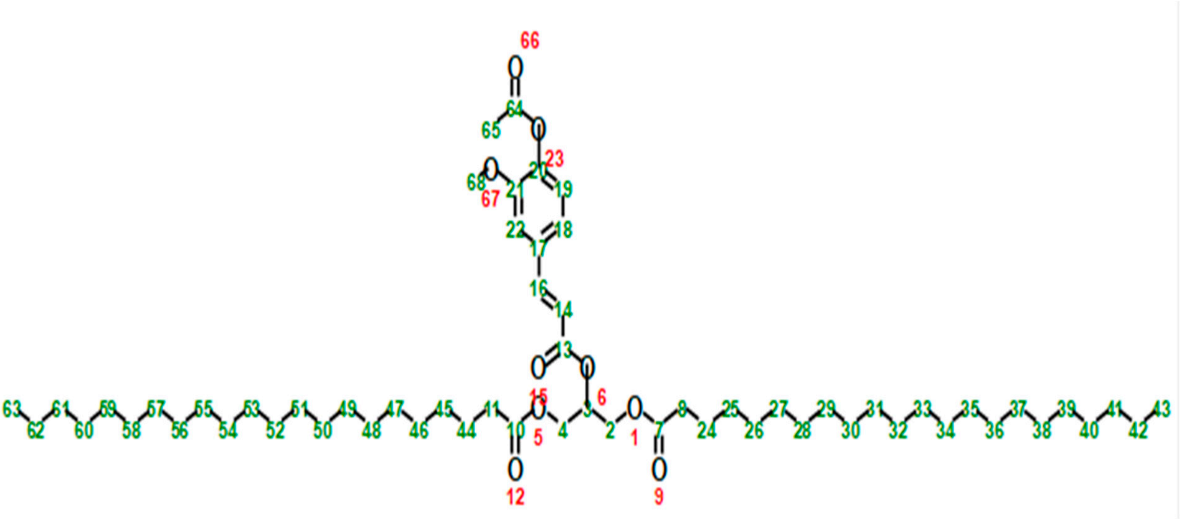

Figure **S42.** Structure enumerated by atoms of the product **12** used for signal assignment in NMR spectra.

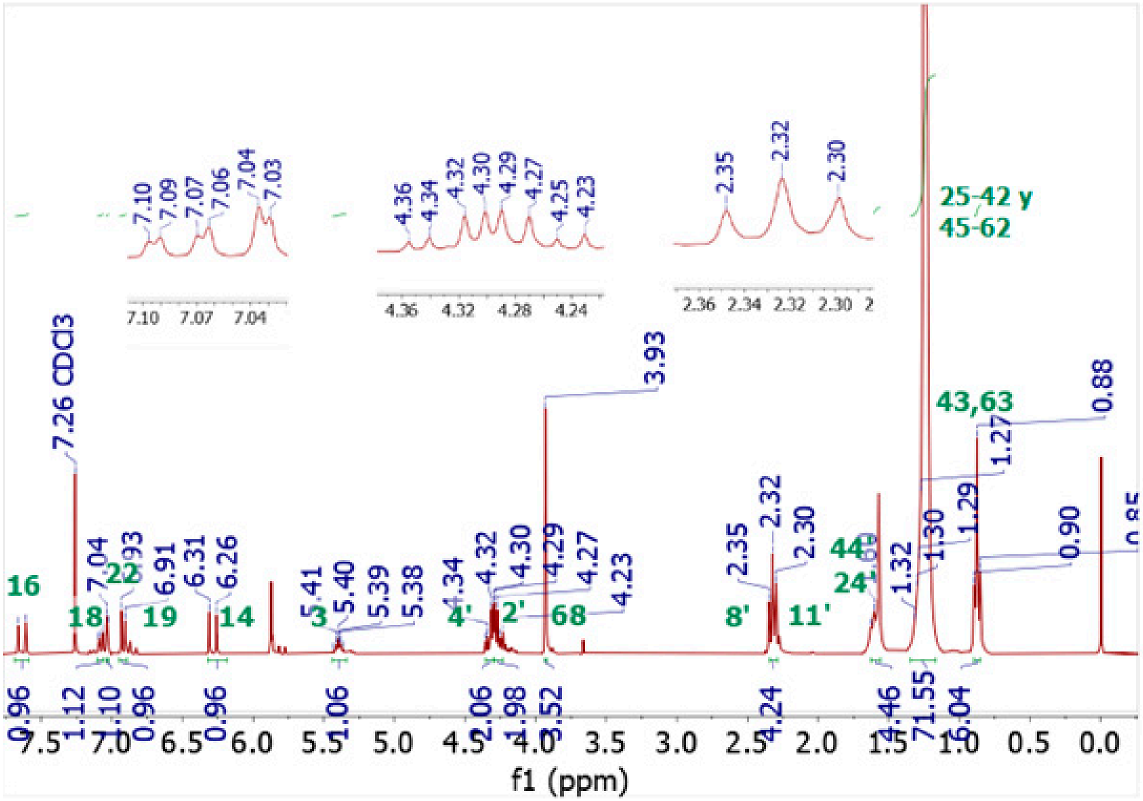

Figure **S43**. ^{1}H NMR Spectra of **12**.

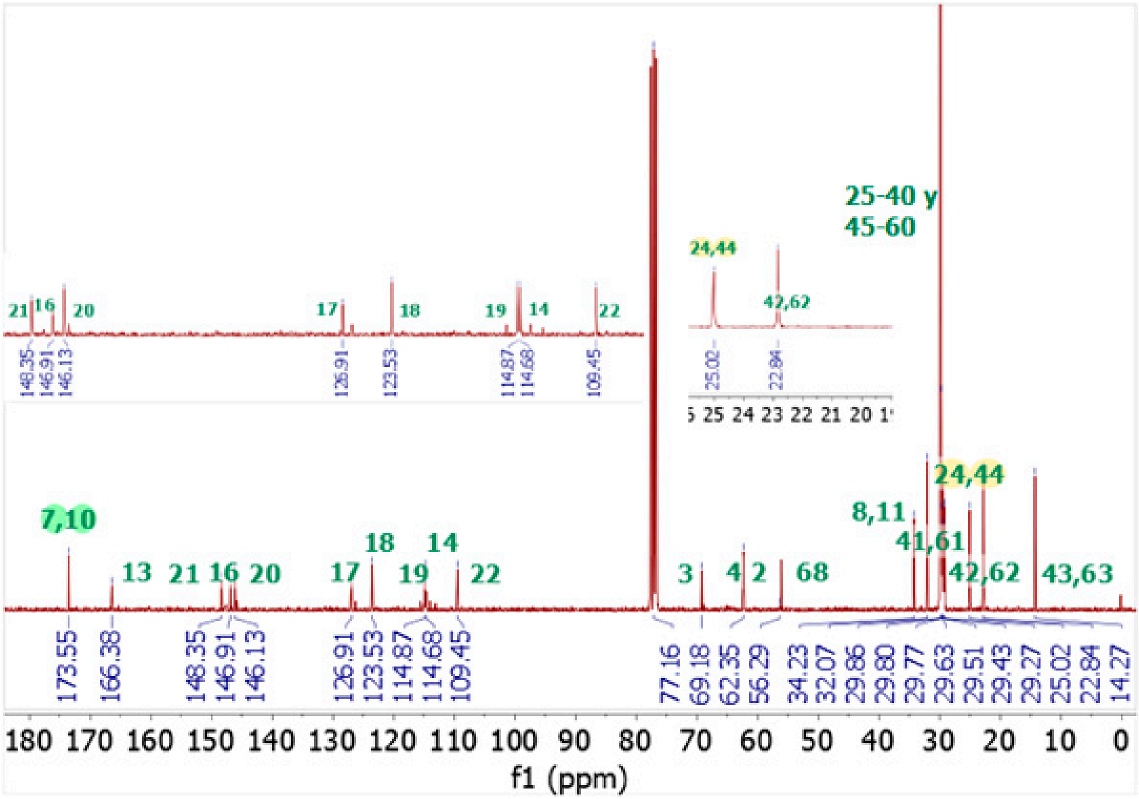

Figure **S44**. ^{13}C NMR Spectra of **12**.

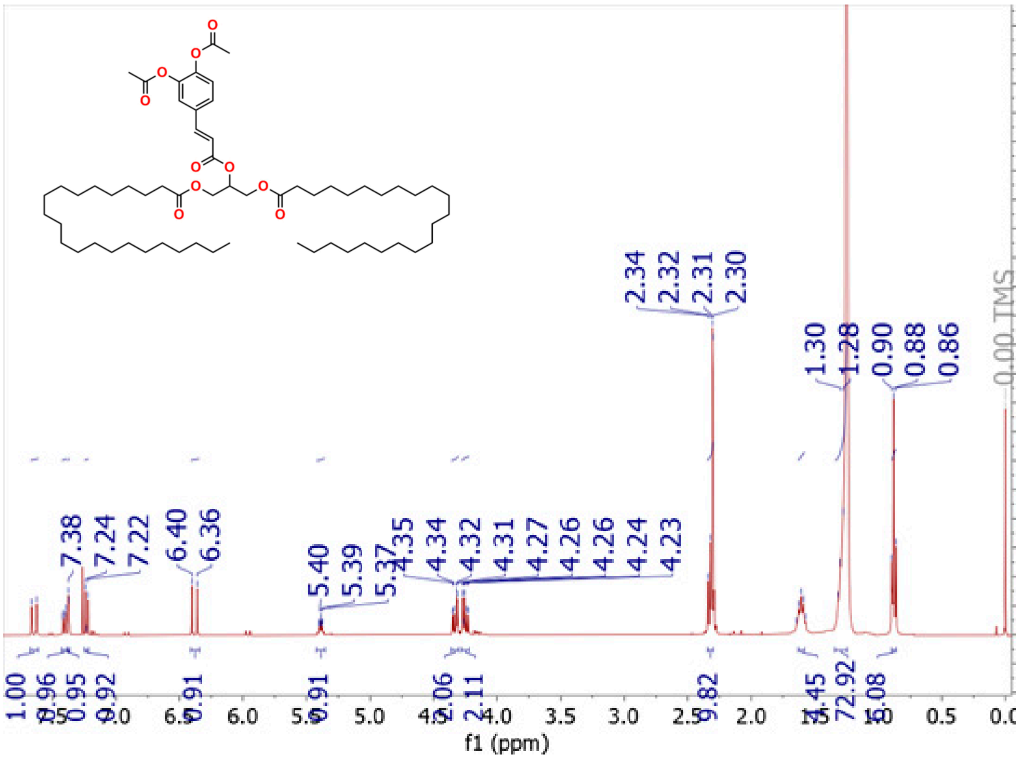

Figure **S45**. ^{1}H NMR Spectra of **14**.

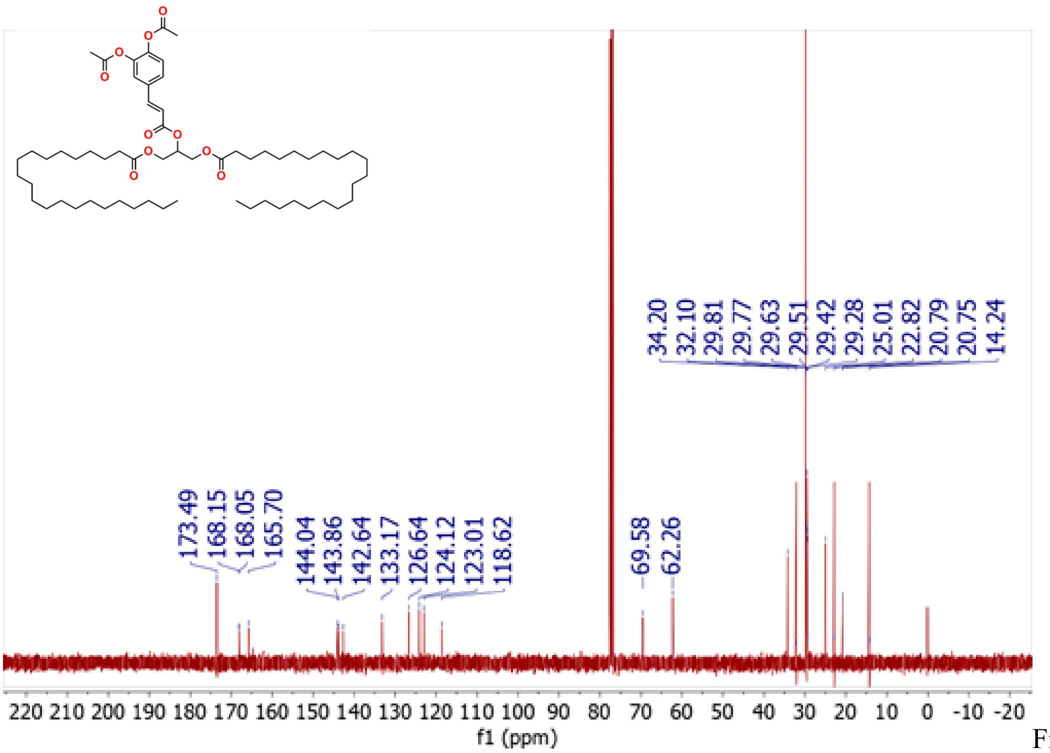

Figure **S46**. ^{13}C NMR Spectra of **14**.

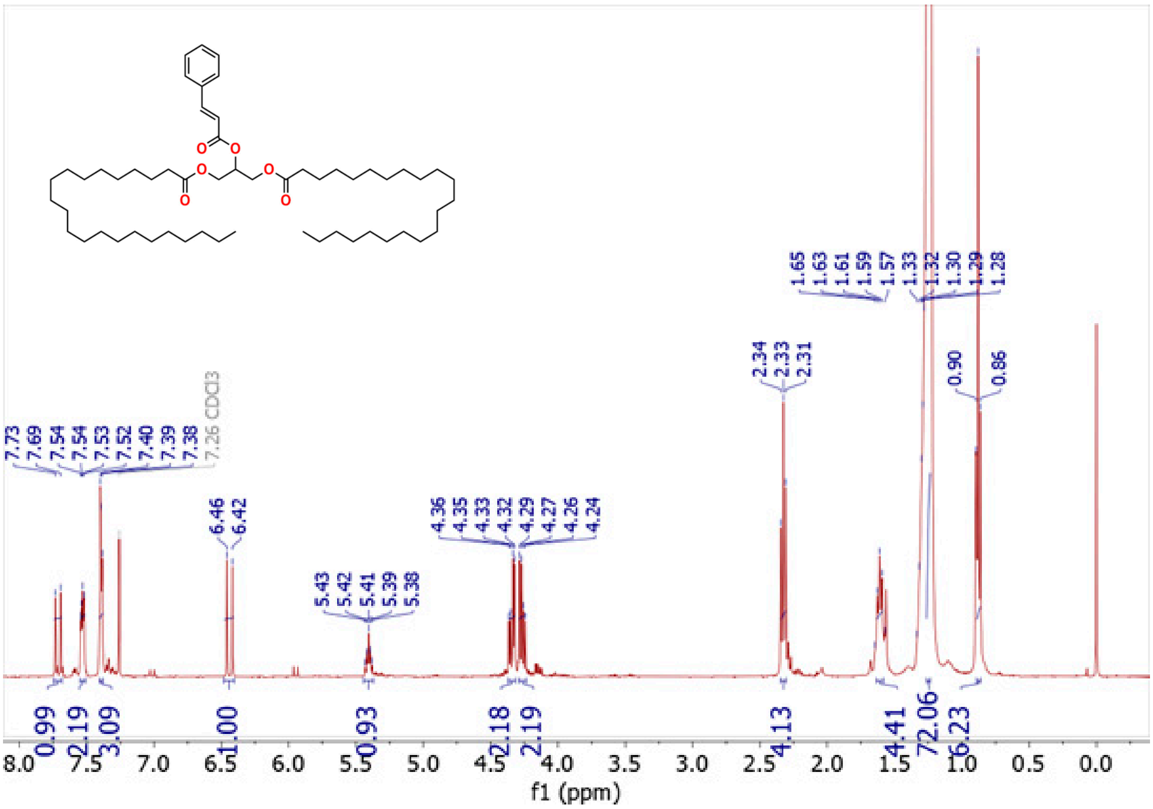

Figure **S47**. ^{1}H NMR Spectra of **15**.

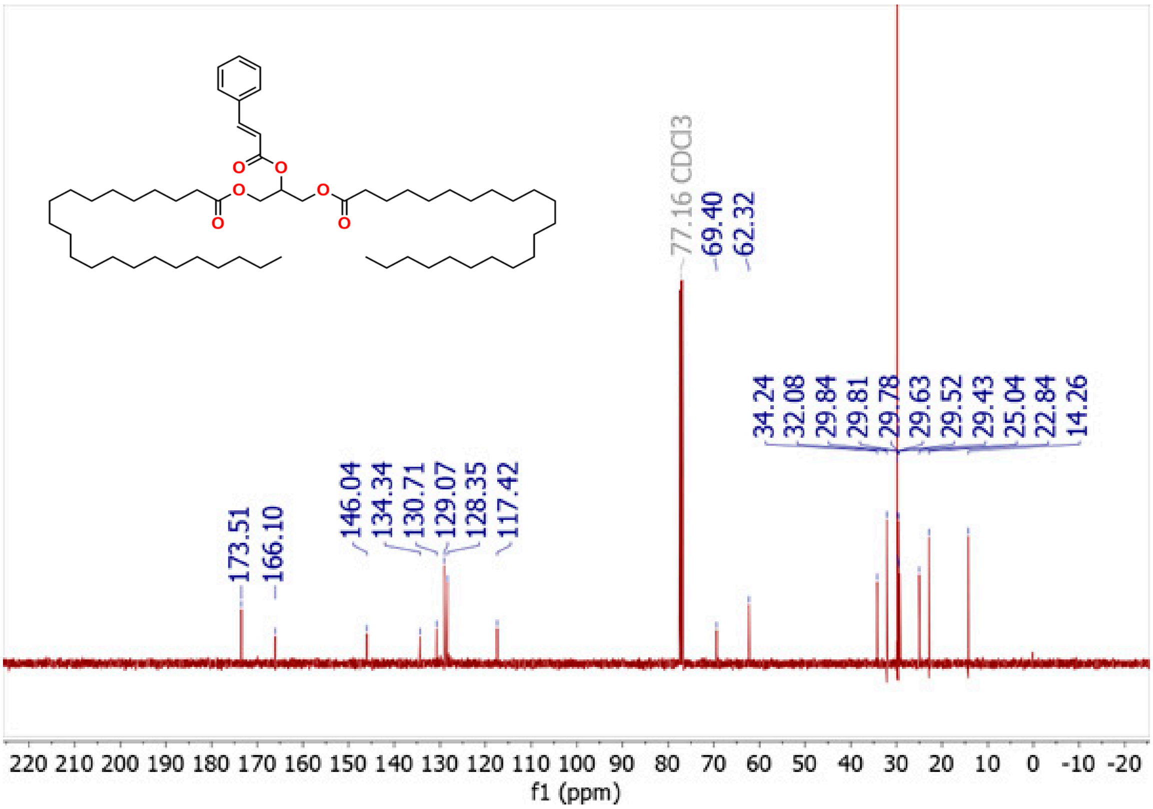

Figure **S48.** ^{13}C NMR Spectra of **15**.

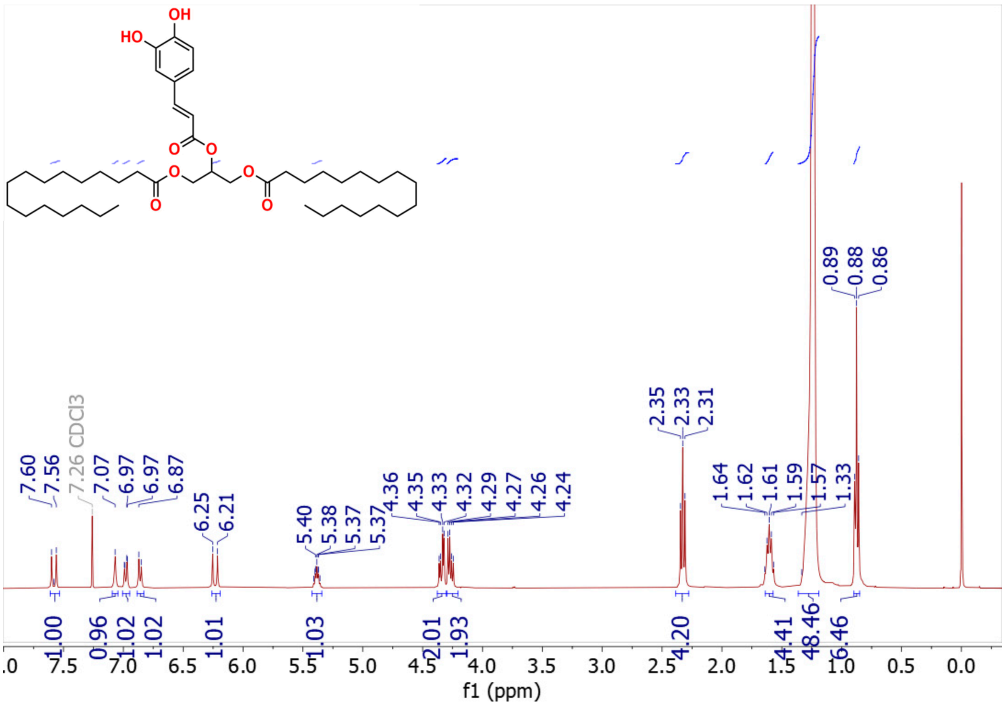

Figure **S49**. ^{1}H NMR Spectra of **19**.

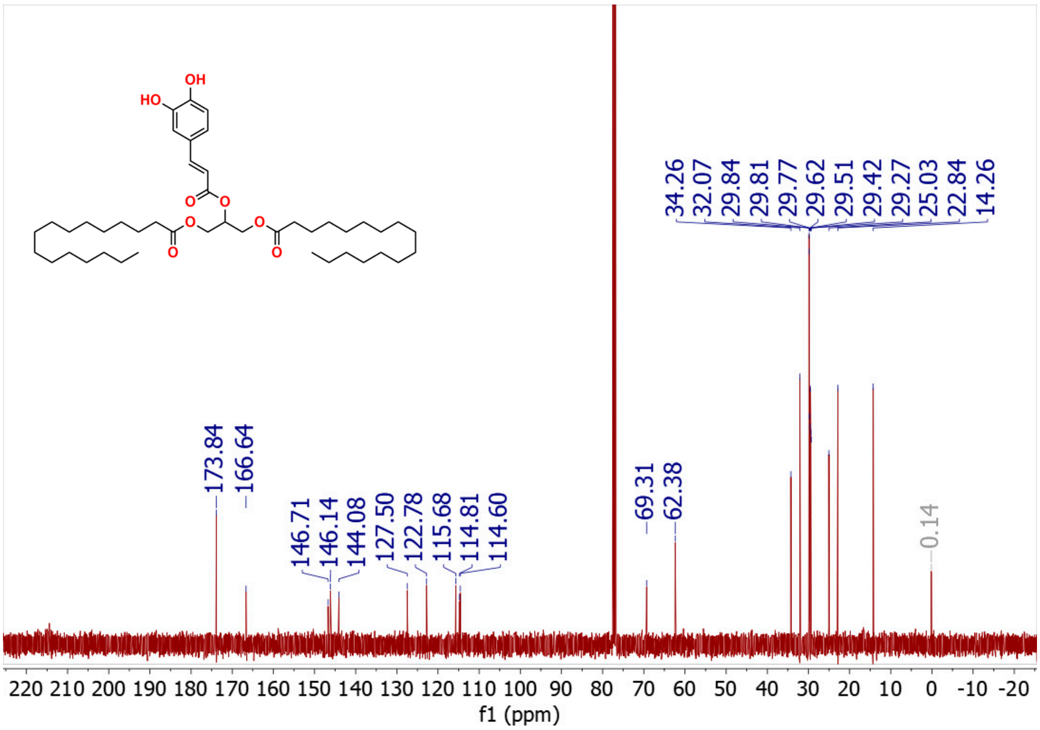

Figure **S50**. ^{13}C NMR Spectra of **19**.

<u>3 Spectra (^{1}H, ^{13}C NMR and MS) of the compound 1 (1,3-dibehenyl-2-feruryl glyceride)...S51-S53.</u>

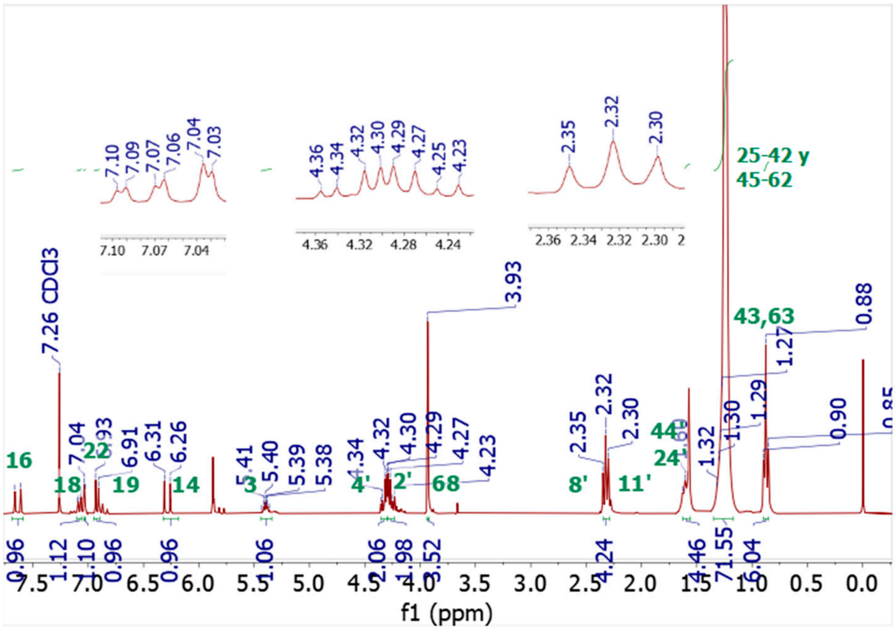

Figure **S51**. ^{1}H NMR Spectra of **1.**

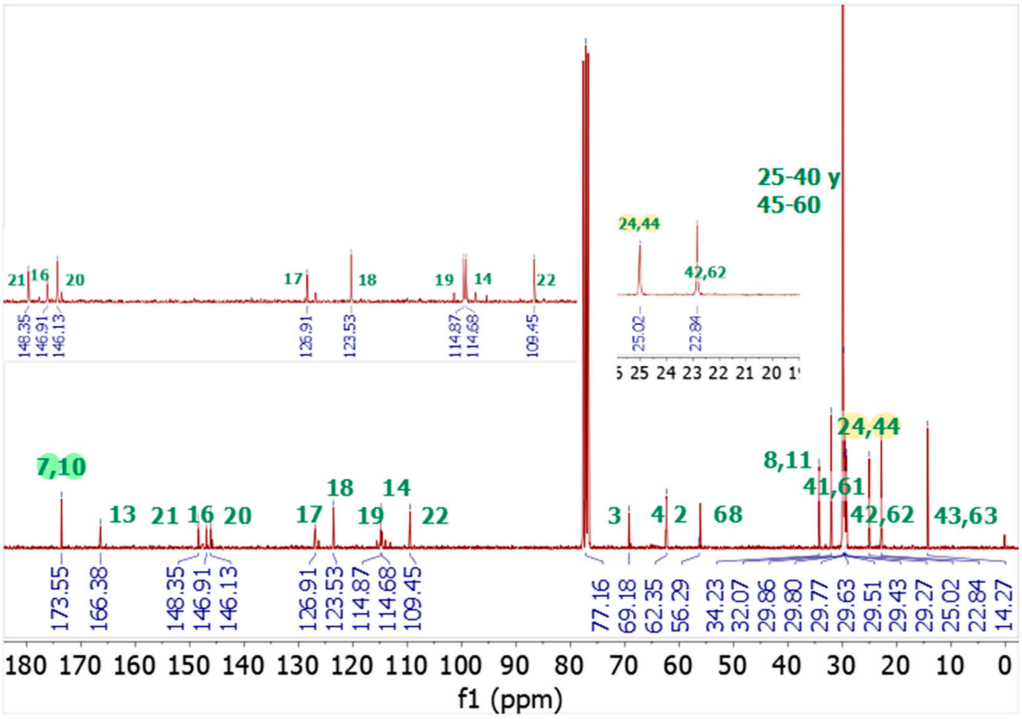

Figure **S52**. ^{13}C NMR Spectra of **1.**

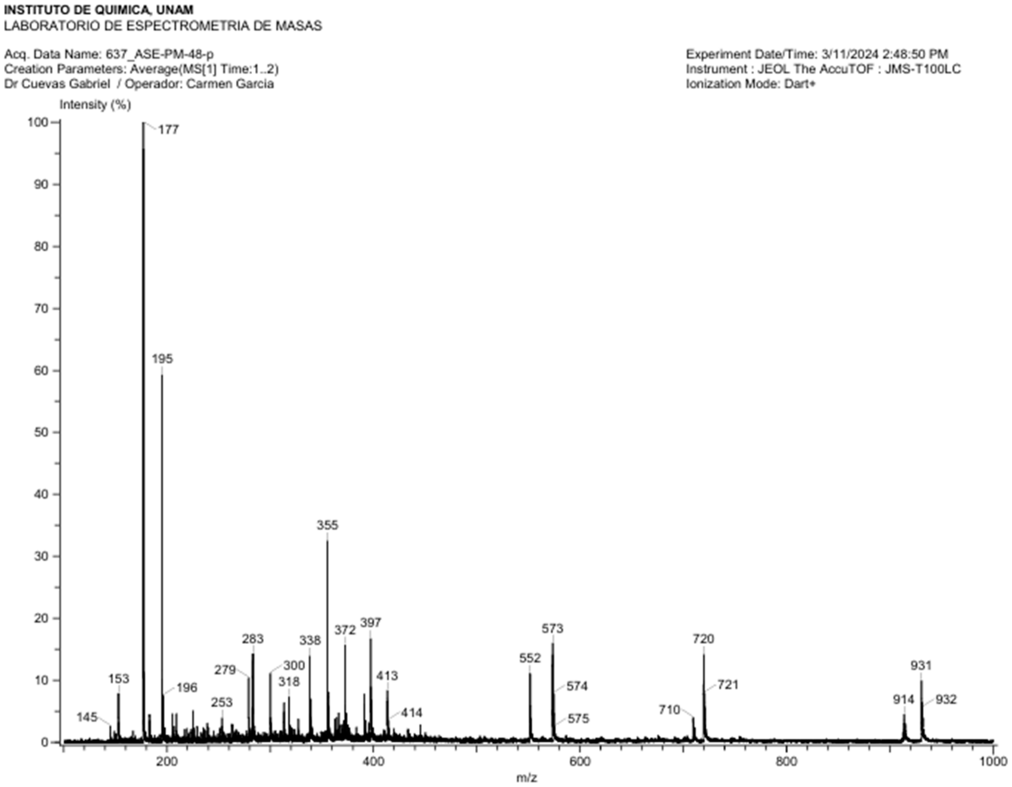

Figure **S53.** DART^{+} spectrum for product **1**(1,3-dibehenyl-2-feruryl glyceride).

Product 1 was analyzed by mass spectrometry using the DART+ (Direct Real-Time Analysis) ionization technique to determine its m/z ratio. The theoretical analysis revealed a molecular weight of 913.4 g for the condensed formula $C_{57}H_{100}O_8$. The spectrum for Product 1 revealed a fragmentation pattern common to structures with linear hydrocarbon chains. The DART+ spectrum for the compound in question is analyzed below:

A peak was found at 914 m/z, corresponding to the molecular ion [M+H], indicating the presence of the desired product. A peak was also found at 931 m/z, corresponding to the hydrated molecular ion [M+H_2O]. To the left of the molecular ion peak, a variety of signals are present, reflecting the nature of the desired molecule. The peak at 720 m/z corresponds to the molecular ion [M-193]; this signal can be attributed to the fragmentation of natural product 1, resulting in the release of the ferulic acid fragment. A signal is also observed at 195 m/z, corresponding to the ferulic acid ion [$M_{ferulic}$+ H]. At 573 m/z, a signal appears, which is due to the fragmentation of product 1, causing the release of a complete behenic acid chain, yielding the molecular ion [M-340]. The peaks at 117 and 195 m/z showed the highest intensity in the spectrum and are explained by the multiple fragmentation of the target molecule, as are the peaks adjacent to those mentioned above. The product exhibits four regions where fragmentation is most likely: the substituents of the aromatic ring (hydroxyl and methoxyl groups) and the aliphatic chains. Due to the high fragmentation of the molecule into different ions, within which peaks corresponding to the raw materials used (1,3-DAG and acetylated ferulic acid) were found, it was decided to perform elemental analysis of the product.

Table S54. Elemental analysis report for the product 1,3-dibehenyl-2-feruryl glyceride and percentage of error obtained for the elemental analysis of 1.

Key to sample:ASE-PM-48-P

Theoretical values: %C = 74.95 % and %H = 11.04

registration number	Trial	C [%]	H [%]	N [%]	date of analysis
62	a	75.22	11.06	---	03 20, 2024
62	b	75.08	11.06	---	03 20 , 2024
---	Average	75.15	11.06	---	

Percentage of error

Product	Theoretical results		Experimental results		percentage of error	
	C [%]	H [%]	C [%]	H [%]	C	H
1	74.95	11.04	75.15	11.06	0.26	0.18

According to the American Chemical Society (ACS) and other reputable institutions, an acceptable error percentage for elemental analysis is less than 0.4%. Therefore, the product matches the theoretical data presented, as the error percentage is below the accepted limit. This confirmed the identity of product **1**.
